# The cell adhesion molecule CD44 acts as a modulator of 5-HT7 receptor functions

**DOI:** 10.1186/s12964-024-01931-0

**Published:** 2024-11-23

**Authors:** Saskia Borsdorf, Andre Zeug, Yuxin Wu, Elena Mitroshina, Maria Vedunova, Supriya A. Gaitonde, Michel Bouvier, Michael C. Wehr, Josephine Labus, Evgeni Ponimaskin

**Affiliations:** 1 Cellular Neurophysiology, Hannover Medical School, Hannover, Germany; 2 Research Group Cell Signalling, Department of Psychiatry and Psychotherapy, LMU University Hospital, LMU Munich, Munich, Germany; 3 Department of Neurotechnology, Institute of Biology and Biomedicine, Lobachevsky University of Nizhni Novgorod, Nizhny Novgorod, Russia; 4 Department of Biochemistry and Molecular Medicine, Institute for Research in Immunology and Cancer (IRIC), Université de Montréal, Montréal, QC Canada; 5 Systasy Bioscience GmbH, Planegg-Martinsried, Germany

**Keywords:** G protein-coupled receptor (GPCR), Receptor oligomerization, Serotonin receptor 7 (5-HT7R), Hyaluronan receptor CD44, Fluorescence Resonance Energy Transfer (FRET), Bioluminescence Resonance Energy Transfer (BRET)

## Abstract

**Background:**

Homo- and heteromerization of G protein-coupled receptors (GPCRs) plays an important role in the regulation of receptor functions. Recently, we demonstrated an interaction between the serotonin receptor 7 (5-HT7R), a class A GPCR, and the cell adhesion molecule CD44. However, the functional consequences of this interaction on 5-HT7R-mediated signaling remained enigmatic.

**Methods:**

Using a quantitative FRET (Förster resonance energy transfer) approach, we determined the affinities for the formation of homo- and heteromeric complexes of 5-HT7R and CD44. The impact of heteromerization on 5-HT7R-mediated cAMP signaling was assessed using a cAMP responsive luciferase assay and a FRET-based cAMP biosensor under basal conditions as well as upon pharmacological modulation of the 5-HT7R and/or CD44 with specific ligands. We also investigated receptor-mediated G protein activation using BRET (bioluminescence resonance energy transfer)-based biosensors in both, homo- and heteromeric conditions. Finally, we analyzed expression profiles for 5-HT7R and CD44 in the brain during development.

**Results:**

We found that homo- and heteromerization of the 5-HT7R and CD44 occur at similar extent. Functionally, heteromerization increased 5-HT7R-mediated cAMP production under basal conditions. In contrast, agonist-mediated cAMP production was decreased in the presence of CD44. Mechanistically, this might be explained by increased Gαs and decreased GαoB activation by 5-HT7R/CD44 heteromers. Unexpectedly, treatment of the heteromeric complex with the CD44 ligand hyaluronic acid boosted constitutive 5-HT7R-mediated cAMP signaling and receptor-mediated transcription, suggesting the existence of a transactivation mechanism.

**Conclusions:**

Interaction with the hyaluronan receptor CD44 modulates both the constitutive activity of 5-HT7R as well as its agonist-mediated signaling. Heteromerization also results in the transactivation of 5-HT7R-mediated signaling via CD44 ligand.

**Supplementary Information:**

The online version contains supplementary material available at 10.1186/s12964-024-01931-0.

## Background

G protein-coupled receptors (GPCRs) represent the largest family of membrane-spanning proteins and are valuable therapeutic targets [1]. They process external stimuli and regulate numerous cellular functions by activating downstream effectors, including heterotrimeric G proteins, which in turn initiate the production of various second messengers [2]. Research over the past decades has shown that GPCRs can form homo- and hetero-dimers as well as higher order oligomers, which can significantly alter the signaling properties, such as GPCR trafficking, ligand binding, and G protein activation and selectivity [3–6].

Receptor oligomerization was also reported for the serotonin receptor family, including the serotonin receptor 7 (5-HT7R), the most recently described serotonergic receptor forming homo- and hetero-oligomers [7, 8]. The 5-HT7R is canonically involved in the production of cAMP via the stimulation of the heterotrimeric Gs protein [9, 10]. In the brain, the 5-HT7R exerts a variety of physiological functions, including learning and memory processing [11, 12]. There is also strong evidence for the involvement of the 5-HT7R in a variety of psychiatric and neurological disorders, such as anxiety, obsessive–compulsive disorder, schizophrenia, epilepsy, and neurodegenerative diseases [13–17]. While the 5-HT7R is known to be activated by the neurotransmitter serotonin (5-HT) and by several synthetic ligands, there is increasing in vitro and in vivo evidence for its high agonist-independent constitutive activity [18–22]. However, the functional consequences of receptor homo- and hetero-oligomerization for the agonist-dependent and independent signaling remain to be elucidated.

Although oligomerization between GPCR family members have been extensively studied, physical interactions of GPCR with non-GPCRs are much less characterized and mainly involve interactions with receptor tyrosine kinases (RTKs) and channel proteins [23–25]. We have previously demonstrated that 5-HT7R can interact with CD44 [26], though the functional consequences of this interaction on 5-HT7R-mediated signaling remain elusive. CD44 is the major receptor for hyaluronan in the brain, a key component of the extracellular matrix (ECM) involved in various aspects of neuronal plasticity [27–29]. CD44 is a single-pass transmembrane protein that is involved in cell adhesion and cytoskeleton modulation [30–33]. We have recently demonstrated that both receptors belong to the same signaling module involving the matrix metalloproteinase-9 (MMP-9) and the small GTPase Cdc42 [26]. Activation of this signaling pathway can modulate dendritic spine remodeling and synaptic transmission in hippocampal neurons [26].

Here, using a quantitative FRET approach we elucidated the efficiency of CD44 and 5-HT7R to form homo- and heteromers. We also applied a FRET-based biosensor to analyze changes in cAMP levels in living cells under basal conditions and after pharmacological modulation of both receptors. Using enhanced bystander BRET, we finally analyzed the role of 5-HT7R/CD44 heteromerization on activation of different classes of heterotrimeric G proteins.

## Methods

### Materials

Murine neuroblastoma N11-115 cells were obtained from the American Type Culture Collection (ATCC®, Cat# CRL-2263, RRID:CVCL_0451) (Virginia, USA). 5-Carboxamidotryptamine maleate (5-CT), and SB-269970 hydrochloride (SB) were obtained from Tocris (Wiesbaden-Nordenstedt, Germany). Hyaluronic acid sodium salt from *Streptococcus equi* sp., (-)-epinephrine ( +)-bitartrate salt, dopamine hydrochloride, 3-isobutyl-1-methylxanthine (IBMX), forskolin (FSK), pertussis toxin, and penicillin/streptomycin were purchased from Merck (Darmstadt, Germany; Oakville, ON, Canada). Dulbecco’s Modified Eagle Medium (DMEM), Lipofectamine 2000, and salmon sperm DNA were obtained from Invitrogen (Darmstadt, Germany; Burlington, ON, Canada) and fetal calf serum (FCS) from Bio&SELL (Feucht, Germany). HEK-293SL (HEK-293 termed from here) were a gift from S. Laporte (McGill University, Montreal, Quebec, Canada) and have been described before [34]. Newborn calf serum (NCS) and penicillin/streptomycin for culturing of HEK-293 cells were obtained from Wisent Inc. (Saint-Jean-Baptiste, QC, Canada). Polyethylenimine (PEI) was purchased from Polysciences, Inc (Warrington, PA, USA) and Prolume Purple from Nanolight™ Technology (Pinetop, AZ, USA). If not stated otherwise, the following concentrations were used: 5-CT (10 µM), SB-269970 (100 nM), hyaluronic acid (20 µg/mL), IBMX (50 µM), FSK (5 µM), pertussis toxin (100 ng/mL).

### Animals

Expression profiles of 5-HT7R and CD44 was performed in male C57BL/6 mice from the following age groups: P1, P5, P15, P30, 6 months, 12 months, 18 months and 22–24 months. Six animals per group were analyzed. Animals were housed at 12 h day/night cycle under SPF conditions. The mice were provided with ad libitum access to food and water. The study was strictly adhered to the fundamental principles governing the care and housing of experimental animals as outlined in the "International Guiding Principles (Code of Ethics) for Biomedical Research Involving Animals" (CIOMS and ICLAS, 2012). The ethical principles established by the European Convention for the protection of vertebrate animals used for experimental and other scientific purposes were also respected (Strasbourg, 2006). All experimental procedures were approved by the Bioethics Committee of Lobachevsky University.

### Plasmids and recombinant DNA procedures

The following expression plasmids were used in the present study: enhanced cyan and yellow fluorescent protein (eCFP, eYFP)-tagged [7] as well as green fluorescent protein (eGFP)-tagged [35] 5-HT7R in the pEGFP vector. Human influenza hemagglutinin (HA)-tagged 5-HT7R [7], as well as eYFP- and eGFP-tagged CD44 [26], and the FRET-based cAMP biosensor [36] in the pcDNA3.1 plasmid. An eCFP-eYFP tandem construct in the pEYFP-N1 plasmid [37] and the FRET-based RaichuEV-Cdc42/KRasCT biosensor in the pPBbsr2 plasmid [38]. Overlap extension polymerase chain reaction (PCR) was used on CD44-eGFP and 5-HT7R-eGFP to generate HA-, mCherry-, and eCFP-tagged CD44 as well as mCherry-tagged 5-HT7R, respectively. CD86-eCFP and CD86-eYFP were kindly gifted by Prof. Dr. Moritz Bünemann, Philipps-Universität Marburg, Germany [39]. For the cAMP responsive element (CRE) luciferase assays, a hRluc (*Renilla reniformis*) luciferase under the control of a CMV promoter cloned into a pGL4.75 vector (Promega, #E6931) and a luc2 firefly luciferase under the control of CRE in a pGL4.16 vector (Addgene plasmid #194,384) were used. BRET biosensors, Gα subunits and constructs expressing β2 adrenergic receptor and dopamine D2 receptor in pcDNA3.1 vectors were described previously [40]. For split TEV plasmids, the HTR7, CD44, and CD86 open reading frame (ORF) sequences were amplified via PCR using the Q5 High-Fidelity DNA Polymerase (NEB), and the resulting PCR was BP-recombined into the pDONR/Zeo plasmid using Gateway recombination cloning (Thermo Fisher Scientific, Waltham, MA, USA). Each entry clone plasmid was control-digested using BsrGI, which cuts inside the recombination sequences and thus released the insert. Lastly, HTR7, CD44, and CD86 ORF sequences were verified via Sanger sequencing. Gateway LR recombination was used to transfer the ORFs from the entry vectors into the split TEV destination vectors (either pcDNA3_attR1-ORF-attR2-NTEV-tcs-GV-2xHA_DEST (Addgene plasmid #194,385) or pcDNA3_attR1-ORF-attR2-CTEV-2xHA_DEST) to yield pcDNA3_HTR7-NTEV-tcs-GV-2xHA, pcDNA3_CD44-CTEV-2xHA, pcDNA3_CD86-NTEV-tcs-GV-2xHA, and pcDNA3_CD86-CTEV-2xHA. For measuring split TEV-based interactions, a reporter plasmid carrying a luc2 firefly reporter gene driven by 10 × clustered upstream activated sequences (10xUAS) linked to a minimal MLP promoter was used (pGL4_10xUAS-MLPmin-luc2, Addgene plasmid #194,383).

### Quantitative Real-Time Polymerase Chain Reaction (qRT-PCR)

Total RNA from the hippocampal and prefrontal cortex brain lysates were isolated using ExtractRNA kit (Evrogen). Transcription was performed using a commercial MMLV RT kit (Evrogen) and expression analysis was executed on a 2720 Thermal Cycler amplifier system (Applied Biosystems). Following primer pairs were used for detection:

OAZ_ fw: TGAGGGCAGTAAGGACAGTTT; OAZ_rev: TTCGGAGTAGGGCGGCTCT.

CD44_fw: GCTTCAATGCCTCAGCCC; CD44_rev: CATCACGGTTGACAATAGTTAT.

Htr7_fw: GACCACCTATCGTAGCCTA; Htr7_rev: GGTCACAGTTTTGTAGCACA.

Calculation of relative mRNA levels was performed using the ∆∆Ct method.

### Cell culture and transfection of N1E-115 cells

Murine N1E-115 neuroblastoma cells were cultured in DMEM supplemented with 10% fetal calf serum and 1% penicillin/streptomycin under humidified conditions at 5% CO_2_ and 37 °C. Transient transfection with vectors was performed using Lipofectamine 2000 according to the manufacturer’s instructions 16 h after seeding. Cells were exposed to 1 µg (12-well) or 0.4 µg (48-well) DNA. For single receptor transfections, the empty vector (pcDNA) was transfected to equalize DNA concentrations. Experiments were performed 12 h post-transfection.

### Cell culture and transfection of HEK-293 cells

HEK-293 cells were cultured in DMEM supplemented with 10% NCS and 1% penicillin/streptomycin. Cells were kept under humidified conditions at 37 °C and 5% CO_2_. For transfection, the DNA (1000 ng/1 mL cell suspension), adjusted with salmon sperm DNA, was diluted in phosphate buffered saline (PBS) and mixed with 3000 ng PEI at a ratio of 1:3. The PEI-DNA mix was incubated for 15 min and then added to the cell suspension. Cells were seeded at a density of 35,000 cells/100 µL/well onto white 96-well plates (Greiner Bio-One). For BRET experiments, cells were cultured for 48 h.

### Confocal laser scanning microscopy

Live-cell imaging experiments were accomplished with a Zeiss LSM 780 equipped with a 40 × /1.2 NA water immersion objective and ZEN 2012 software. General measurement settings included a bit depth of 16 bit at 1024 × 1024 pixels or 512 × 512 pixels for lux-FRET and Cdc42 measurements or cAMP measurements, respectively. Before measurement, N1E-115 were transferred from cell culture medium to osmolality-adjusted tyrode buffer (15 mM NaCl, 1 mM KCl, 0.1 mM MgCl_2_, 0.2 mM CaCl_2_, 1 mM HEPES, pH 7.4, glucose for osmolality adjustment).

### Measurement of cAMP responses

cAMP dynamics were measured using a FRET-based biosensor [36] which shows a decreased FRET efficiency and acceptor/donor (Citrine/mCerulean) ratio upon increasing cAMP concentrations (Fig. 2A). Time-series measurements of cAMP levels were performed according to Prasad et al. (2019). In brief, N1E-115 cells were seeded onto 18 mm glass coverslips and transfected with the receptors and the cAMP biosensor as described. Images were obtained every 10 s for 10 min (60 cycles à 10 s) with continuous focus correction using the Zeiss “Definite Focus”. The cAMP biosensor and eGFP-coupled receptors were excited with a laser beam of 440 nm. Spectral separation of fluorophores was obtained with the Zeiss “Online Fingerprinting Mode” after recording background-corrected spectra of the single fluorophores. Signals of eGFP- and mCherry-tagged receptors were obtained using the “Lambda Mode” and spectra were unmixed using custom-written MatLab scripts. For equilibration, measurements of cells were performed for three minutes before stimulation. The mean of the measured cAMP biosensor ratio values obtained during this equilibration phase were used to calculate the baseline ratio of the cAMP sensor. Stimulants were applied via a perfusion system (Warner Instruments, Hamden, CT, USA) with a perfusion rate of 3 mL/min. Blocking reagents (i.e. SB-269970 and pertussis toxin) were applied prior to the measurements. Ratio-time curves were obtained on a pixel-based approach for each cell separately and were fitted to the following exponential fit model [35]:

$$y=A\left(1-{e}^{-\frac{t-{t}_{0}}{\tau }}+polyoff\right)$$

where *A* is the response amplitude, *τ* the response time, *t* the time, *t*_*0*_ the time-point of treatment, and *y* the biosensor ratio. The calculated response amplitudes *A* are reported as negative amplitudes that correlate positively with the accumulation of cAMP.

### Measurement of Cdc42 activity

To investigate activation of the small GTPase Cdc42, N1E-115 cells were seeded onto 18 mm-coverslips and transfected with mCherry-coupled CD44 and the FRET-based Raichu-Cdc42 (RaichuEV-Cdc42/KRasCT), which shows an increased YPet-to-mTurquoise ratio upon Cdc42 activation (Supplementary Figure S4A) [38]. Time-series measurements were performed with continuous focus correction using the Zeiss “Definite Focus” with image acquisition every 2.5 min. Baseline Cdc42 activity was obtained for 7.5 min, followed by the stimulation and subsequent measurement for another 15 min. Excitation of YPet and mTurquoise were performed at 440 nM and of mCherry at 561 nm. Ratio-time curves were obtained on a pixel-based approach for each cell separately using custom-written MatLab scripts.

### Lux-FRET measurements

To calculate FRET efficiencies for eCFP- and eYFP-tagged receptors, N1E-115 cells were subjected to lux-FRET measurements 24 h post-transfection. Live-cell imaging measurements were performed as described before [35]. Briefly, z-stacks were obtained at 440 nm and 514 nm excitation capturing a spectrum ranging from 410–695 nm. Data were evaluated as described above. As control, monomeric eCFP- and eYFP-tagged CD86 was used [39]. Data were obtained from five independent experiments with five technical replicates each.

To estimate affinities for homo- or heteromerization, N1E-115 cells were transfected with varying donor and acceptor concentrations for 5-HT7R homomers (5-HT7R-eCFP/5-HT7R-eYFP), CD44 homomers (CD44-eCFP/CD44-eYFP) as well as 5-HT7R-CD44 heteromers (5-HT7R-eCFP/CD44-eYFP vs CD44-eCFP/5-HT7R-eYFP). Twenty-four hours post-transfection, cells were detached from the cell culture dish using a pre-warmed tyrode buffer (15 mM NaCl, 1 mM KCl, 0.1 mM MgCl2, 0.2 mM CaCl2, 1 mM HEPES, pH 7.4). Cell suspensions were analyzed in the fluorescence spectrometer Fluorolog-3.22 (Horiba) equipped with a xenon lamp (450 W, 950 V) and two double monochromators. The following configuration and settings were used: 10 mm pathway quartz cuvettes at room temperature in "front face" arrangement, dual excitation 440 nm and 488 nm, with emission spectra 450 – 600 nm and 498 – 600 nm, respectively, 0.5 s integration time. The spectral contributions from light scattering and nonspecific fluorescence of the cells were taken into account by subtracting the emission spectra of water and non-transfected cells (background) from each measured spectrum, respectively. Samples were measured as technical duplicates from three independent experiments. Donor-to-acceptor ratio was calculated using a custom-written MatLab script.

### Lux-FRET calculations

To determine the apparent FRET efficiency for 5-HT7R homomers, CD44 homomers, and 5-HT7R/CD44 heteromers, we used lux-FRET method that has been described in detail previously [7, 37]. This approach allows calculation of the total concentration ratio [A^t^]/[D^t^] of donor and acceptor and donor molar fraction *x*_D_ = [D^t^]/([D^t^] + [A^t^]), containing the relative concentrations of the donor [D^t^] and the acceptor [A^t^]. Additionally, the apparent FRET efficiencies *Ef*_D_ and *Ef*_A_, where the FRET efficiency *E* is weighted by the fractions of donors (*f*_D_ = [DA]/[D^t^]) and acceptors (*f*_A_ = [DA]/[A^t^]) in complexes, respectively, can be determined. Calculations were performed as described previously. In brief, two-emission spectra were obtained for the receptor pairs, reference cells containing the fluorophores separately, and a tandem construct expressing the fluorophore pair at a fixed ratio of 1:1. Fitting of obtained spectra and calculations of apparent FRET efficiencies and donor molar fractions were performed with custom-written MATLAB scripts.

### Calculation of kinetic constants

For the calculation of the relative dissociation constants describing the homo- and hetero-oligomerization processes, we applied a previous published dimerization model [7] on the lux-FRET data obtained with the spectrofluorometer. This model allows determining individual relative dissociation constants *K* for the different oligomerization types in an equilibrium state.

Briefly, the apparent FRET efficiencies were plotted against the *x*_D_ values and the data was fitted by finding a numerical solution of Equation 5 from Renner et al. [7] For the fit, minimization of the weighted least square differences was applied. Measurements with *x*_D_ values lower than 0.1 and higher than 0.9 were excluded from the calculations, since FRET efficiencies for these donor mole fractions show high systemic errors. Additionally, individual *E* values were allowed for the homo- and heteromers. The error of the fit was calculated according to ${\chi }^{2}= \sum_{i=1}^{n}{({y}_{i}-f\left({x}_{i}\right))}^{2}/f({x}_{i})$ where *y*_*i*_ are the measured apparent FRET efficiencies and *f(x*_*i*_*)* are the values obtained by the fit.

### G protein effector membrane translocation (GEMTA) assay

To test Gα protein activation the BRET-based (G)EMTA was performed [40]. Therefore, HEK-293 cells were transfected with the receptor of interest, rGFP-CAAX, the Gα subunit (Gαs(67)-RlucII for Gαs), and for the Gαi/o family additionally with Rap1GAP-RlucII. On the day of the BRET assay, cells were washed with PBS and then equilibrated in tyrode (140 mM NaCl, 2.7 mM KCl, 1 mM CaCl_2_, 12 mM NaHCO_3_, 5.6 mM D-glucose, 0.5 mM MgCl_2_, 0.37 mM NaH_2_PO_4_, 25 mM HEPES [pH 7.4]) at 37 °C and 10% CO_2_ for at least 30 min. Cells were stimulated with the agonists at different concentrations for 5 min. Afterwards, the luciferase substrate Prolume Purple (1.3 µM, Nanolight™ technologies) was added and cells were incubated for another 5 min. BRET signals were measured with a Spark® multimode plate reader (Tecan) equipped with filters covering 515 ± 20 nm for the acceptor and 400 ± 70 nm for the donor. BRET^2^ values were calculated by dividing intensity values emitted from the acceptor (rGFP, 515 nm) by the intensity values measured for the donor (RlucII, 410 nm). Dose–response curves were fitted to a three-parameter logistic nonlinear regression model and calculated minimum values and the span were used as baseline BRET^2^ ratios and response amplitudes, respectively.

### CRE luciferase reporter gene assay

CRE-driven gene expression was analyzed using a luciferase reporter gene assay (PJK) according to the manufacturer’s protocol. Briefly, N1E-115 cells were transfected simultaneously with the CRE-driven Firefly luciferase luc2, the CMV-driven *Renilla* luciferase hRluc, and the HA-tagged receptors. 24 h after transfection, cells were stimulated for 6 h before cell lysis. Cell lysates were subjected to a black 96-well plate (Perkin Elmer, Rodgau, Germany). The addition of luciferase substrates and the measurement of light emission, were performed with a Berthold Mithras LB 940 Multimode Plate Reader. The protocol included injection of the substrate, a 2 s shaking period, and acquisition for 0.5 s. The addition of the substrates and measurement of the emission was carried out first for the *Renilla* and then for the Firefly luciferase. Obtained emission values for the CRE-driven luc2 were normalized to the CMV-driven hRluc luciferase as an internal control.

### Split TEV luciferase assays

The performance of split TEV protein–protein interaction assays is described in detail by Wu et al. [41]. In brief, assays were conducted in flat-bottom 96-well clear plates (Falcon) using 2 × 10^4^ HEK-293 cells plated per well. Cells were transfected with split TEV plasmids, i. e., both NTEV and CTEV plasmids, and the reporter plasmid pGL4_10xUAS-MLPmin-luc2. Per plasmid, 10 ng were used. Plasmids were transfected using the Turbofect reagent (Thermo Fisher Scientific) at a ratio of 1 µg of DNA to 3 µL of Turbofect. After 20 h post transfection, cells were lysed with 30 µL of passive lysis buffer (Promega, Madison, WI, USA). Firefly luciferase activity was measured with a Mithras LB 940 Microplate Reader (Berthold Technologies, Bad Wildbad, Germany) using the MicroWin 2000 software.

### Statistics

Statistical analyses and curve fitting were performed using GraphPad Prism versions 8.0.1 and 10.0.3. Performed statistical tests and number of independent experiments (*N*) are indicated in the figure legends. For biosensor experiments, fold values to matched control groups are reported to eliminate high variances in biosensor responses and baseline values and to report only changes between groups. One-sample t-tests were used to test for difference from a mean of one. *p* values < 0.05 were considered statistically significant. For normally distributed multiple comparisons, one- or two-way analysis of variance (ANOVA) were performed. Post hoc tests after ANOVA were only performed if overall ANOVA F-test achieved *p* < 0.05 and no significant inhomogeneity in variance were detected. *P* values > 0.05 were considered not significant. For multiple comparisons, adjusted *p* values < 0.05 were considered statistically significant.

## Results

### 5-HT7R and CD44 form homo- and heteromeric complexes

**Fig. 1:**
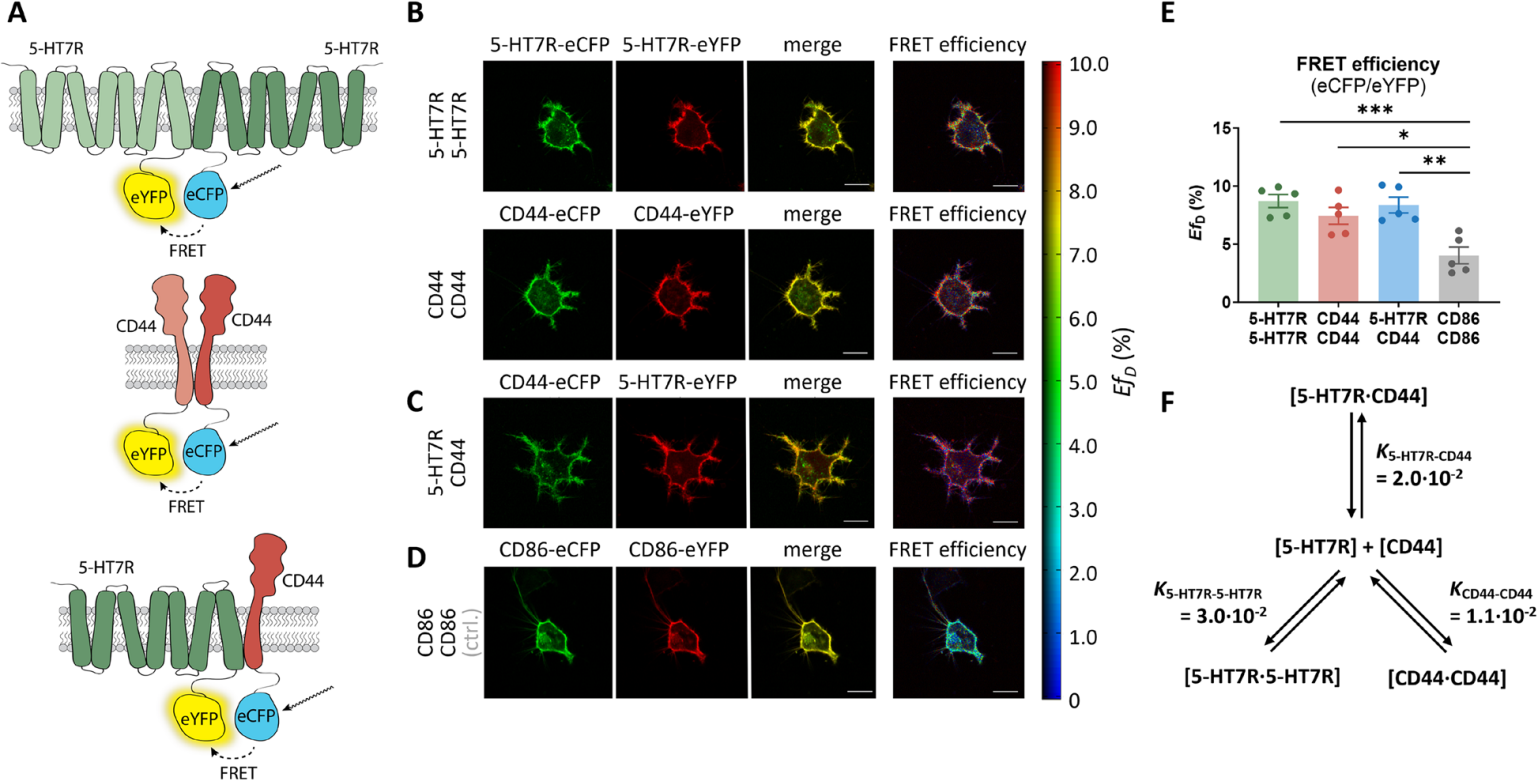
5-HT7R and CD44 form homo- and heteromeric complexes with similar probabilities. **A** Schematic representation of the experimental design. **B**-**D** Representative images of N1E-115 cells co-expressing 5-HT7R-eCFP/5-HT7R-eYFP (**B**, upper panel) or CD44-eCFP/CD44-eYFP (**B**, lower panel), CD44-eCFP/5-HT7R-eYFP (**C**) or CD86-eCFP/CD86-eYFP (**D**) depicted in eCFP: green, eYFP: red, merge: yellow. The right images show apparent FRET efficiencies (E*f*_D_) between eCFP and eYFP fluorophores. Scale bar: 20 µm. **E** Apparent FRET efficiencies (E*f*_D_) for 5-HT7R- and CD44-homomers, 5-HT7R/CD44-heteromers, and CD86 monomers. Data are means ± SEM (*N* = 5 experiment days, 41–45 cells). Statistical significance was assessed by one-way ANOVA with post hoc Tukey’s multiple comparison test (**E**). * *p* < 0.05, ** *p* < 0.01, *** *p* < 0.001. **F** Dimerization model of 5-HT7R and CD44. Relative dissociation constants *K* were computed from lux-FRET measurements (see Supp. Fig. S1 C-E) using a dynamic oligomerization model [7]

It has been previously demonstrated that both 5-HT7R and CD44 can form homodimers [7, 8, 42–44]. Our recent study suggested that these proteins can also form heteromers [26]. To investigate which kind of complexes (i.e., homo- or heteromers) are preferentially formed, we applied the linear unmixing Förster resonance energy transfer (lux-FRET) approach [37]. To analyze the oligomerization behavior in living cells, N1E-115 cells were transfected with 5-HT7R and CD44 fused to either enhanced cyan (eCFP, donor) or yellow (eYFP, acceptor) fluorescent proteins (Fig. 1A). Both receptors co-localized at the plasma membrane (Fig. 1B, C) and showed a significantly increased apparent FRET efficiency compared to the monomeric control CD86 (Fig. 1B-E). The physical interaction between the 5-HT7R and CD44 was further confirmed by the protein complementation-based split TEV assay [45]. Here, the N-terminal and C-terminal fractions of the NIa protease from the tobacco etch virus (TEV) were fused to the receptors of interest. Upon interaction of the receptors, the TEV protease becomes complemented leading to the release of the transcriptional coactivator GAL4-VP16 (GV), which in turn drives the expression of a firefly luciferase (Supplementary Fig. S1A). Using this approach, we detected a strong luciferase activity upon the expression of the 5-HT7R-NTEV and CD44-CTEV constructs, suggesting a direct interaction between these receptors (Supplementary Fig. S1B). In contrast, the split TEV assay showed significantly lower luciferase activity for cells expressing 5-HT7R-NTEV/CD86-CTEV or CD86-NTEV/CD86-CTEV, further confirming a specific interaction between 5-HT7R and CD44.

Lux-FRET is an intensity-based quantitative FRET approach [37] that provides information on the apparent FRET efficiencies of the donor (*Ef*_D_) and the acceptor (*Ef*_A_), where *f*_D_ and *f*_A_ are the relative amounts of donor and acceptor in oligomeric complexes, respectively. In addition, the donor mole fraction is provided by lux-FRET, which represents the ratio of donor to acceptor molecules (see Methods section for details). With the information about the relative concentrations of receptors in the monomeric and oligomeric state under equilibrium conditions, relative dissociation constants can be calculated, which describe the association behavior. Here, low values correlate with a higher tendency to form oligomers. To this end, we used a general dimerization model that describes the apparent FRET efficiency as a function of the donor mole fraction and the relative dissociation constants [7] and measured the apparent FRET efficiencies at different donor mole fractions. From fitting the experimental data to a general dimerization model (Supplementary Fig. S1 C-F), we determined relative dissociation constants in the following order: K_5-HT7R-5-HT7R_ > K_5-HT7R-CD44_ > K_CD44-CD44_ (Fig. 1F). Since all values are in the same order of magnitude, these data suggest similar affinities for the formation of 5-HT7R and CD44 homo- and heteromers.

### Heteromerization with CD44 modulates the constitutive 5-HT7R activity towards cAMP signaling

**Fig. 2:**
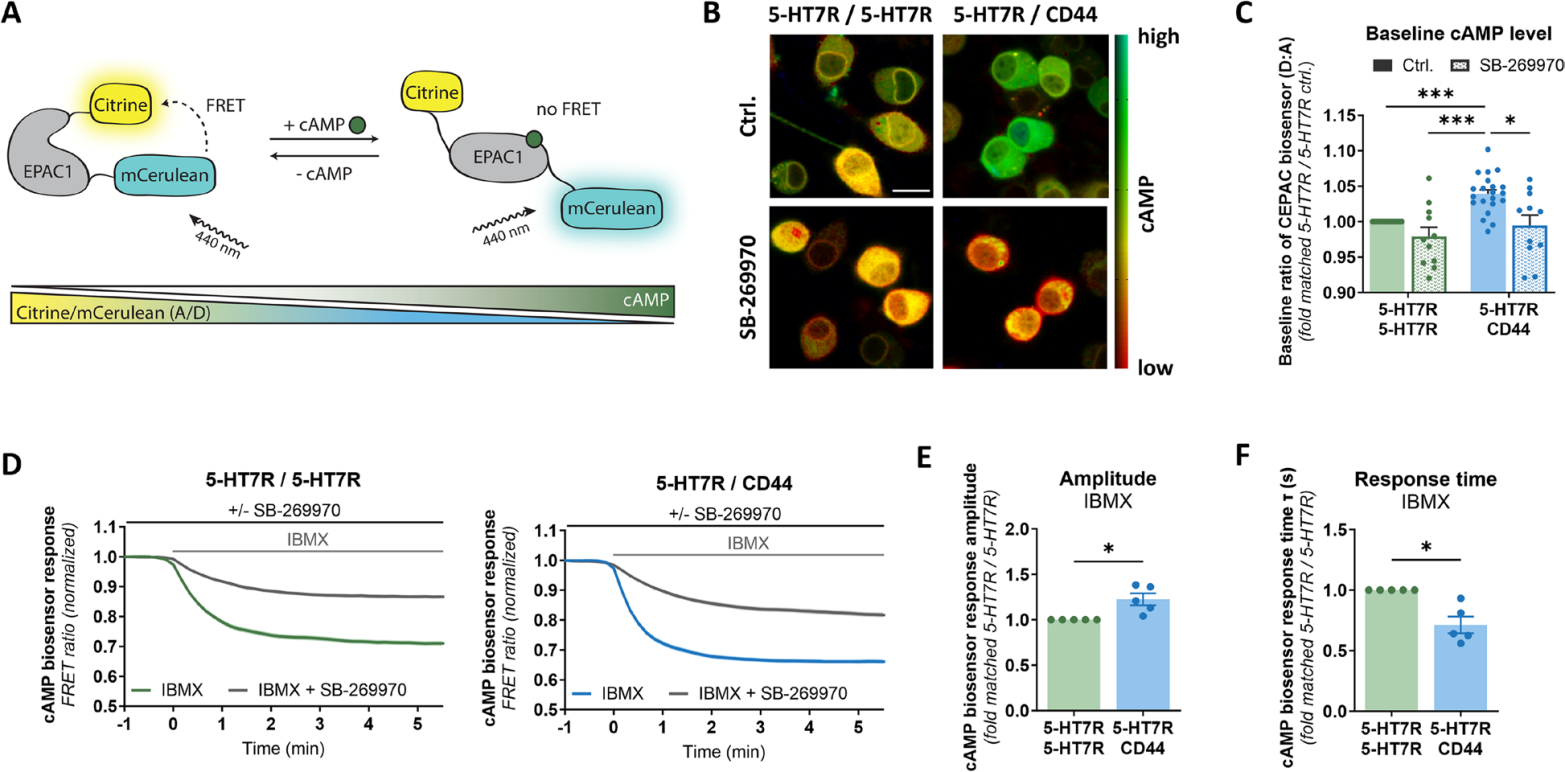
Constitutive 5-HT7R activity is regulated by its heteromerization with CD44. **A** Schematic presentation of the FRET-based cAMP biosensor that consists of EPAC1 (*exchange factor directly activated by cAMP 1*) connected to the fluorophores Citrine (acceptor) and mCerulean (donor). An increase in intracellular cAMP correlates with a decrease in the FRET efficiency and the fluorescence ratio of Citrine/mCerulean. **B**, **C** Representative images (**B**) and quantification (**C**) of the baseline cAMP levels as measured with the cAMP biosensor in N1E-115 cells transiently expressing 5-HT7R-eGFP/5-HT7R-mCherry or 5-HT7R-eGFP/CD44-mCherry without (Ctrl.) and with pre-blocking of the 5-HT7R with the inverse agonist SB-269970 (100 nM). Data are normalized to values obtained for 5-HT7R/5-HT7R. Data are means ± SEM (*N* = 21 experiment days, ≥ 61 coverslips, ≥ 1502 cells). Statistical significance was assessed by Kruskal–Wallis test with post hoc Dunn's multiple comparisons test. * *p* < 0.05, *** *p* < 0.001. **D** Changes in the Citrine/mCerulean ratio of the cAMP biosensor in response to blockage of cAMP degradation by phosphodiesterase inhibitor 3-Isobutyl-1-methylxanthine (IBMX, 50 µM). Responses to IBMX after pre-blocking with the 5-HT7R selective inverse agonist SB-2669970 (100 nM) are also shown. Basal values of the fluorescence ratio were normalized to one. Data are means ± SEM (*N* ≥ 3 experiment days, ≥ 6 coverslips, ≥ 131 cells). **E**, **F** Amplitude (**E**) and response time (**F**) of the cAMP biosensor response upon application of IBMX to cells expressing 5-HT7R-homomers and 5-HT7R/CD44-heteromers. Values were calculated by fitting the response-time curves to an exponential fit model (see Methods section). Data are shown relative to 5-HT7R homomers. Data are means ± SEM (*N* = 5 experiment days, 8 coverslips, ≥ 166 cells). Statistical significance was assessed by one sample t-test. * *p* < 0.05

To assess whether the interaction with CD44 influences 5-HT7R-mediated signaling, we measured 5-HT7R-evoked cAMP production using a FRET-based cAMP biosensor [36], which exhibits a decreased acceptor to donor (i.e., A/D or Citrine/mCerulean) ratio with increasing cAMP levels (Fig. 2A). In N1E-115 cells, we co-expressed the biosensor with the eGFP- and mCherry-tagged receptors representing either homomeric (5-HT7R/5-HT7R and CD44/CD44) or heteromeric (5-HT7R/CD44) conditions and measured the response of the cAMP biosensor at the single cell level (Supplementary Fig. S2A). The highly overlapping emission spectra of the four fluorophores (Supplementary Fig. S2B) were unmixed using the fingerprinting mode (see Methods section).

The 5-HT7R is known to possess a high constitutive activity towards cAMP signaling [18, 21, 22]. Therefore, we first analyzed whether the presence of CD44 modulates the basal activity by measuring the ratio of the cAMP biosensor under non-stimulated equilibrium conditions. We found that in the presence of CD44, the basal cAMP level was markedly increased compared to homomeric 5-HT7R complexes (Fig. 2B, C). Moreover, application of the 5-HT7R-specific inverse agonist, SB-269970 [16], abolished this effect indicating a 5-HT7R-mediated effect. The latter was supported by the observation that expression of homomeric CD44 complexes did not affect the baseline levels of cAMP, neither in presence nor in absence of SB-269970 (Supplementary Fig. S2C, D). In the next step, we measured the time-dependent cAMP accumulation after blocking cAMP degradation by treatment of N1E-115 cells with the phosphodiesterase inhibitor IBMX (3-isobutyl-1-methylxanthine). The cAMP biosensor A/D ratio decreased after the application of IBMX in cells expressing either 5-HT7R alone or in combination with CD44, which reflects an cAMP increase (Fig. 2D). In contrast, cells expressing pcDNA, or CD44, or cells treated with the 5-HT7R inverse agonist SB-269970 showed almost no response to IBMX (Fig. 2D, Supplementary Fig. S2E) highlighting that the cAMP accumulation upon IBMX is caused by the constitutive activity of the 5-HT7R. After fitting the obtained response-time curves to a single-exponential fit model (see Methods section), we found a significantly increased response amplitude and reduced response kinetics in cells expressing 5-HT7R and CD44 heteromeric complexes compared to cells expressing the 5-HT7R alone (Fig. 2E, f). Both, the decreased basal ratio of the cAMP biosensor as well as increased and accelerated cAMP accumulation after IBMX administration confirm an increased basal cAMP level suggesting boosted constitutive activity of the 5-HT7R in the presence of CD44.

### Heteromerization influences agonist-mediated 5-HT7R signaling

**Fig. 3:**
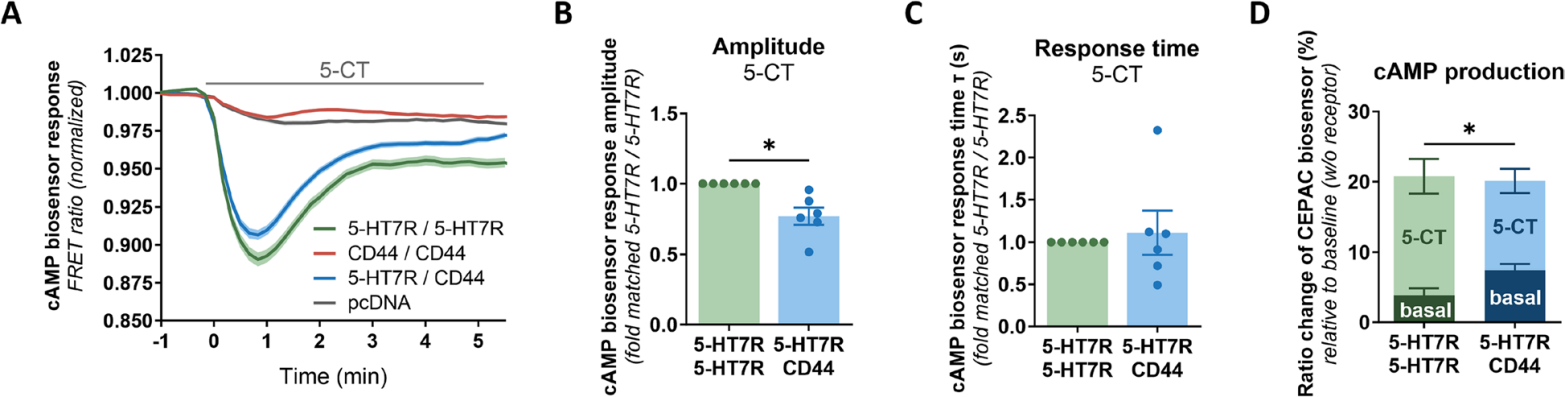
Heteromerization affects agonist-induced 5-HT7R-mediated cAMP production. **A** Traces showing changes in the fluorescence ratio (Citrine/mCerulean) of the cAMP biosensor in response to treatment with the 5-HT7R agonist 5-Carboxamidotryptamine (5-CT, 10 µM). Results are presented for N1E-115 cells co-expressing 5-HT7R-eGFP/5-HT7R-mCherry, CD44-eGFP/CD44-mCherry, 5-HT7R-eGFP/CD44-mCherry, or pcDNA. Basal values of the fluorescence ratio were normalized to one. Data are means ± SEM (*N * ≥ 3 experiment days, ≥ 8 coverslips, ≥ 138 cells). **B**, **C** cAMP biosensor response amplitudes (**B**) and response times (**C**) after treatment with the 5-HT7R agonist 5-CT, compared between 5-HT7R-homodimers (5-HT7R/5-HT7R) and 5-HT7R/CD44-heteromers (5-HT7R/CD44). Amplitude and response time values were calculated after fitting the response-time curves to an exponential fit model (see Methods section). Data are shown relative to 5-HT7R homomerization. Data are means ± SEM (*N* = 6 experiment days, ≥ 12 coverslips, ≥ 278 cells). Statistical significance was assessed by one sample t-test. * *p* < 0.05. **D** cAMP production presented as the percentage change in the cAMP biosensor ratio compared to the basal levels in cells transfected with an empty vector. Changes in response to the expression of 5-HT7R-homomers and 5-HT7R/CD44-heteromers as well as stimulation with 5-CT are depicted. Data presented here are a combination of Figs. 2C and 3B. Data are means ± SEM (*N* = 6 experiment days, ≥ 12 coverslips, ≥ 278 cells). Statistical significance was assessed by two-way ANOVA testing for interaction between the effects of oligomer type (i.e., homo- vs. heteromers) and condition (i.e., basal vs. agonist-induced) on cAMP production. * *p* < 0.05

In the next step, we analyzed the effect of 5-HT7R-CD44 heteromerization on the agonist-induced activation of the 5-HT7R. N1E-115 cells were transfected with either 5-HT7R alone or in combination with CD44, and the cAMP response was measured after treatment of cells with 5-HT7R-specific agonist 5-carboxamidotryptamine (5-CT). Upon stimulation of cells expressing the 5-HT7R with 5-CT, we observed a strong and fast decrease in the acceptor/donor ratio of the cAMP biosensor indicating receptor-evoked cAMP production (Fig. 3A). This effect was 5-HT7R-specific, because it was blocked by application of SB-269970, which also acts as a highly selective 5-HT7R antagonist [46] (Supplementary Fig. S3A, B). When 5-HT7R was co-expressed with CD44, we observed a significantly reduced response amplitude without changes in response kinetics (Fig. 3A-C), demonstrating that heteromerization results in a reduced agonist-induced 5-HT7R activation. Of note, the maximal cAMP response calculated as a sum of responses obtained for the constitutive and the agonist-induced activity was quite similar for both homo- and heteromeric conditions (Fig. 3D). Of note, application of forskolin at the end of measurement resulted in additional increase of cAMP production, demonstrating that the biosensor was saturated neither upon 5-CT nor IMBX treatment (Supplementary Fig. S3C,D). This data suggests that the maximal 5-HT7R-mediated cAMP response is not influenced by receptor heteromerization with CD44. Consequently, increased basal activity of the 5-HT7R participating in heteromeric complexes results in a decreased amplitude of agonist-mediated cAMP response evoked by activation of 5-HT7R (Fig. 3D).

### Stimulation of CD44 within the 5-HT7R/CD44 heteromers results in transactivation of 5-HT7R

**Fig. 4:**
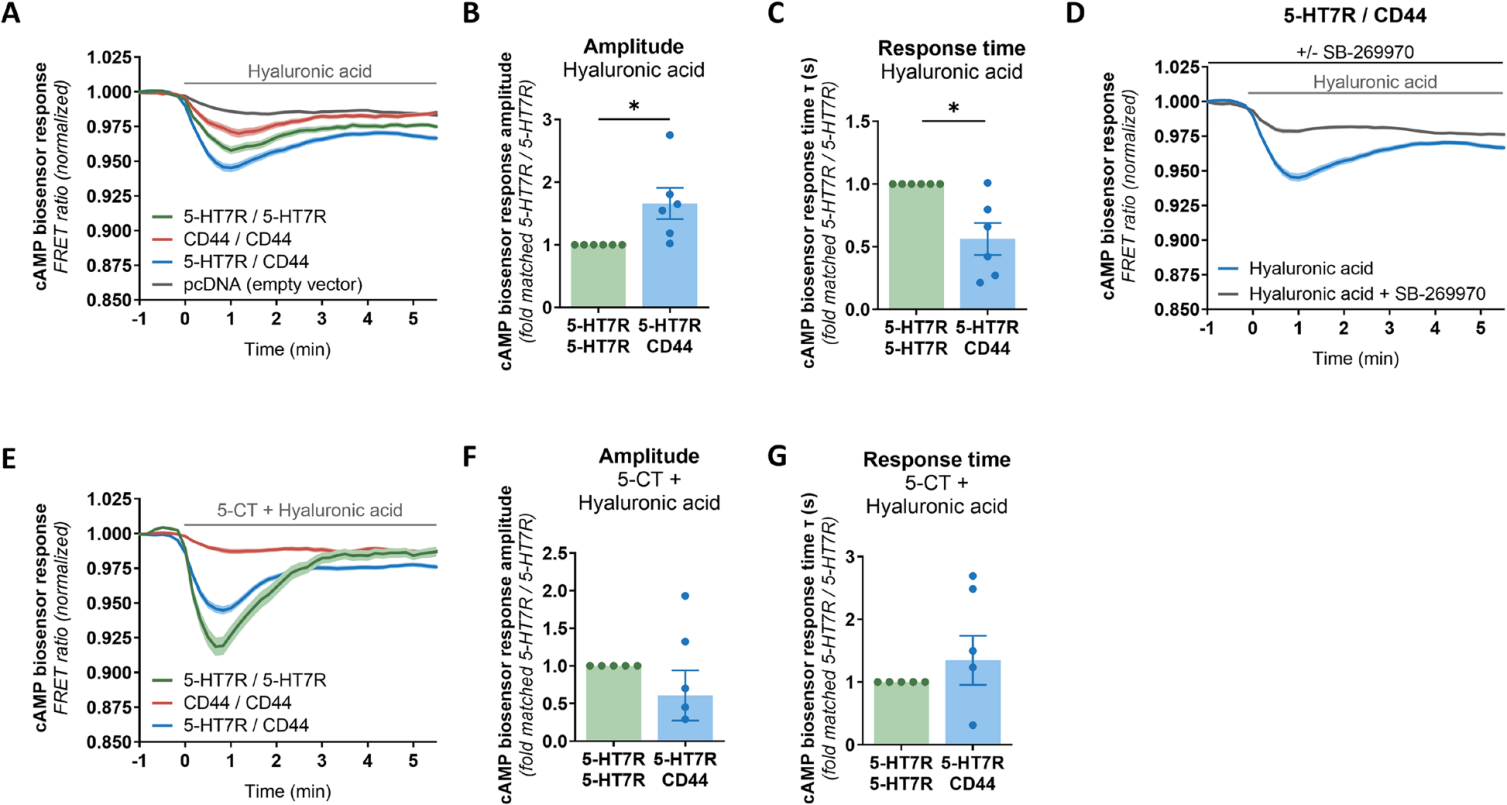
The CD44 agonist hyaluronic acid transactivates the 5-HT7R. **A**-**C** Time-course (**A**) of the cAMP biosensor response upon treatment with the CD44 ligand hyaluronic acid (20 µg/mL). Data are means ± SEM (*N* ≥ 4 experiment days, ≥ 8 coverslips, ≥ 160 cells). Response amplitudes (**B**) and response times (**C**) were calculated from the respective response-time curves. N1E-115 cells were transfected as indicated. Data are means ± SEM (*N* = 6 experiment days, 8 coverslips, ≥ 225 cells). Statistical significance was assessed by one sample t-test. * *p* < 0.05. **D** cAMP response after application of hyaluronic acid (20 µg/mL) in the presence or absence of SB-269970, measured in N1E-115 cells expressing 5-HT7R-eGFP/CD44-mCherry. Data are means ± SEM (*N* ≥ 4 experiment days, 8 coverslips, ≥ 141 cells). **E**–**G** Traces of the cAMP biosensor ratio (**E**) as well as the calculated response amplitudes (**F**) and times (**G**) after simultaneous application of 5-CT and hyaluronic acid. For response-time curves, basal values were normalized to one. Amplitudes and response times are shown relative to 5-HT7R homomers. Data are weighted means ± SEM (*N * ≥ 3 experiment days, ≥ 6 coverslips, ≥ 82 cells (**E**), *N* = 5 experiment days, 8 coverslips, ≥ 134 cells (**F**, **G**)). The square root of the number of measured cells per experimental day was used as the weighting factor. Statistical significance was assessed by one sample t-test. * *p* < 0.05

We next investigated whether the 5-HT7R-mediated signaling can be modulated by stimulating CD44 with its natural ligand, hyaluronic acid. First, we confirmed potent CD44 activation upon hyaluronan stimulation by measuring the inhibition of the CD44 downstream effector Cdc42 using a FRET-based Raichu-Cdc42 biosensor (Supplementary Fig. S4) [47]. In contrast, treatment of these cells with the 5-HT7R agonist 5-CT did not result in any changes of Cdc42 activity (Supplementary Fig. S4). For the activation of 5-HT7R, cAMP production was used as a readout. When CD44 was expressed alone, we did not observe any significant changes in cAMP levels after hyaluronic acid treatment (Fig. 4A). Interestingly, when CD44 was co-expressed with 5-HT7R, treatment with hyaluronic acid resulted in a significant increase in the amplitude of cAMP accumulation, which was dose-dependent with the maximal amplitude obtained at 20 µg/ml concentration of hyaluronan and accompanied by a faster cAMP response kinetics (Fig. 4A-C, Supplementary Fig. S5). This effect was mediated by the 5-HT7R because pre-treatment with the 5-HT7R-specific antagonist SB-269970 blocked this effect (Fig. 4D). Of note, when hyaluronic acid was applied simultaneously with 5-CT, the decrease in the cAMP biosensor response amplitude obtained in case of 5-HT7R/CD44 heteromers after treatment with 5-CT only (Fig. 3B) disappeared, so that we did not observe any differences between 5-HT7R/5-HT7R and 5-HT7R/CD44 conditions after combined HA and 5-CT treatment (Fig. 4E-G). This suggests that stimulation of CD44 with HA leads to the transient transactivation of 5-HT7R rising the cAMP response amplitude. In contrast, the constitutive activity of the 5-HT7R was not affected by the treatment with hyaluronic acid (Supplementary Fig. S6).

**Fig. 5:**
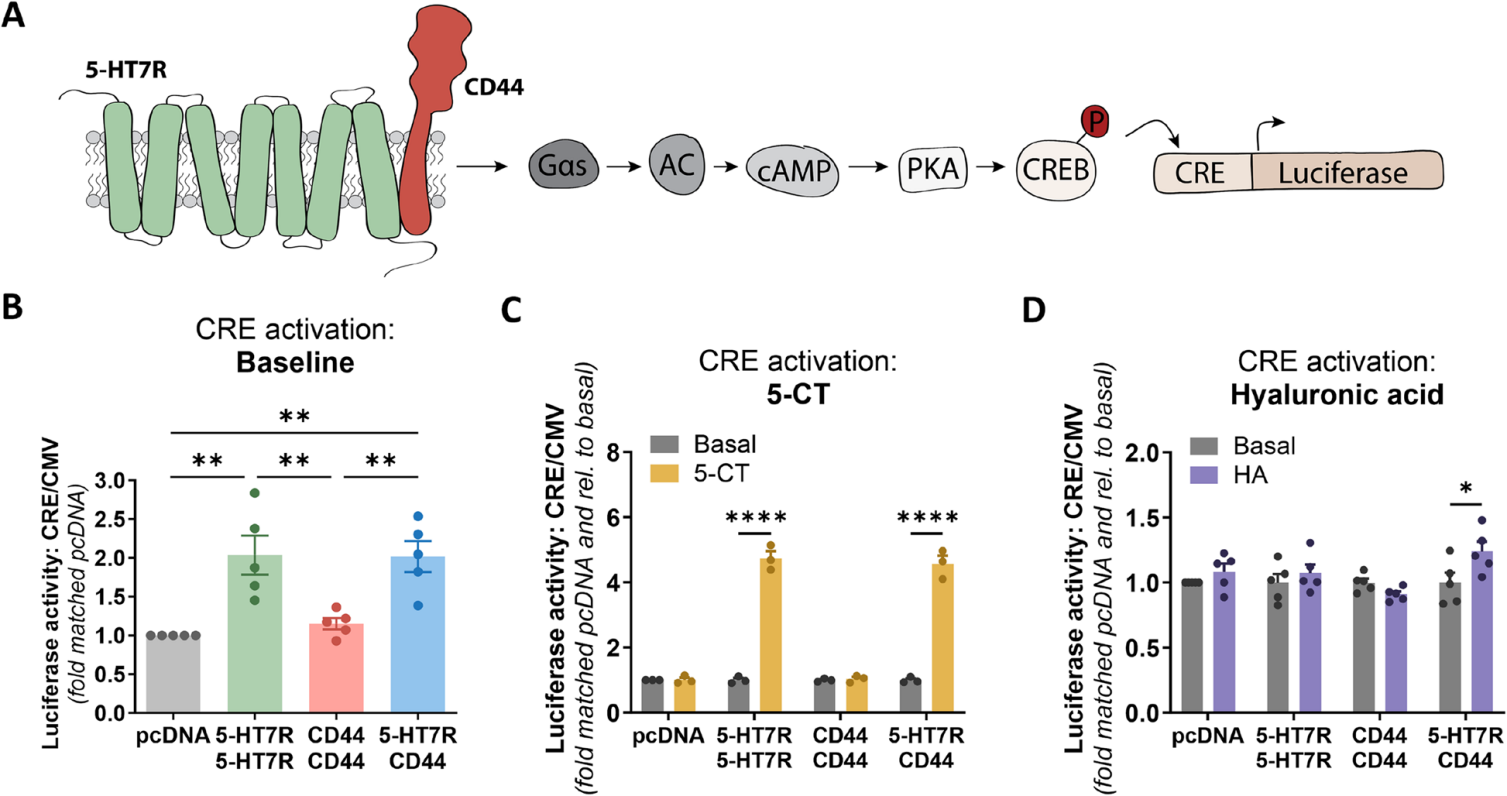
Heteromerization modulates 5-HT7R-mediated gene transcription. **A** Scheme of the luciferase gene reporter assay. Upon production of cAMP, protein kinase A (PKA) gets activated and phosphorylates the cAMP response element binding protein (CREB). Phosphorylated CREB binds to the cAMP response element (CRE) and induces the expression of a luciferase. **B**-**D** Basal (**B**), 5-CT induced (**C**), and hyaluronic acid induced (**D**) activity of CRE-dependent luciferase in N1E-115 cells expressing 5-HT7R-, CD44-homomers, and 5-HT7R/CD44-heteromers. Data are means ± SEM (*N* = 5 experiment days, 15 technical replicates (**B**, **D**), *N* = 3 experiment days, 9 technical replicates (**C**)). Statistical significance was assessed by one-way ANOVA with post hoc Tukey’s multiple comparison test (**B**) or by two-way ANOVA with post hoc Šídák's multiple comparisons test (**C**, **D**; basal vs. treatment). * *p* < 0.05, ** *p* < 0.01, **** *p* < 0.0001

Having demonstrated that heteromerization modulates signaling properties of 5-HT7R towards the cAMP production, we next investigated whether such changes have an impact on the downstream effectors. To address this question, we used the luciferase gene reporter assay in neuroblastoma N1E-115 cells (Fig. 5A). The production of cAMP activates protein kinase A (PKA), which in turn phosphorylates the cAMP response element-binding protein (CREB). The phosphorylated CREB can then bind to the cAMP response element (CRE) to promote the transcription of downstream genes (in our case expression of a firefly luciferase cloned behind the CRE [48]). In line with a high constitutive activity of 5-HT7R, we obtained activation of the cAMP-PKA-CREB pathway as assessed by the doubling of luciferase activity after expression of 5-HT7R alone (Fig. 5B). A similar effect was observed after co-expression of 5-HT7R and CD44 (Fig. 5B). Treatment with 5-CT resulted in about fivefold increase in luciferase activity over the basal levels with no significant difference between homo- and heteromeric complexes (Fig. 5C) suggesting that heteromerization-mediated changes in cAMP production (Figs. 2C and 3B) might be balanced at the level of transcriptional regulation. More importantly, we observed a significant increase in luciferase activity upon treatment of cells co-expressing 5-HT7R and CD44 with hyaluronic acid (Fig. 5D). In contrast, for cells expressing either only 5-HT7R or CD44 no effects on luciferase activity have been observed after hyaluronic acid treatment. These results demonstrate that hyaluronic acid-mediated transactivation of 5-HT7R not only boosts cAMP production but also leads to activation of CRE transcription factor.

### Heteromerization selectively regulates activation of defined G proteins

**Fig. 6:**
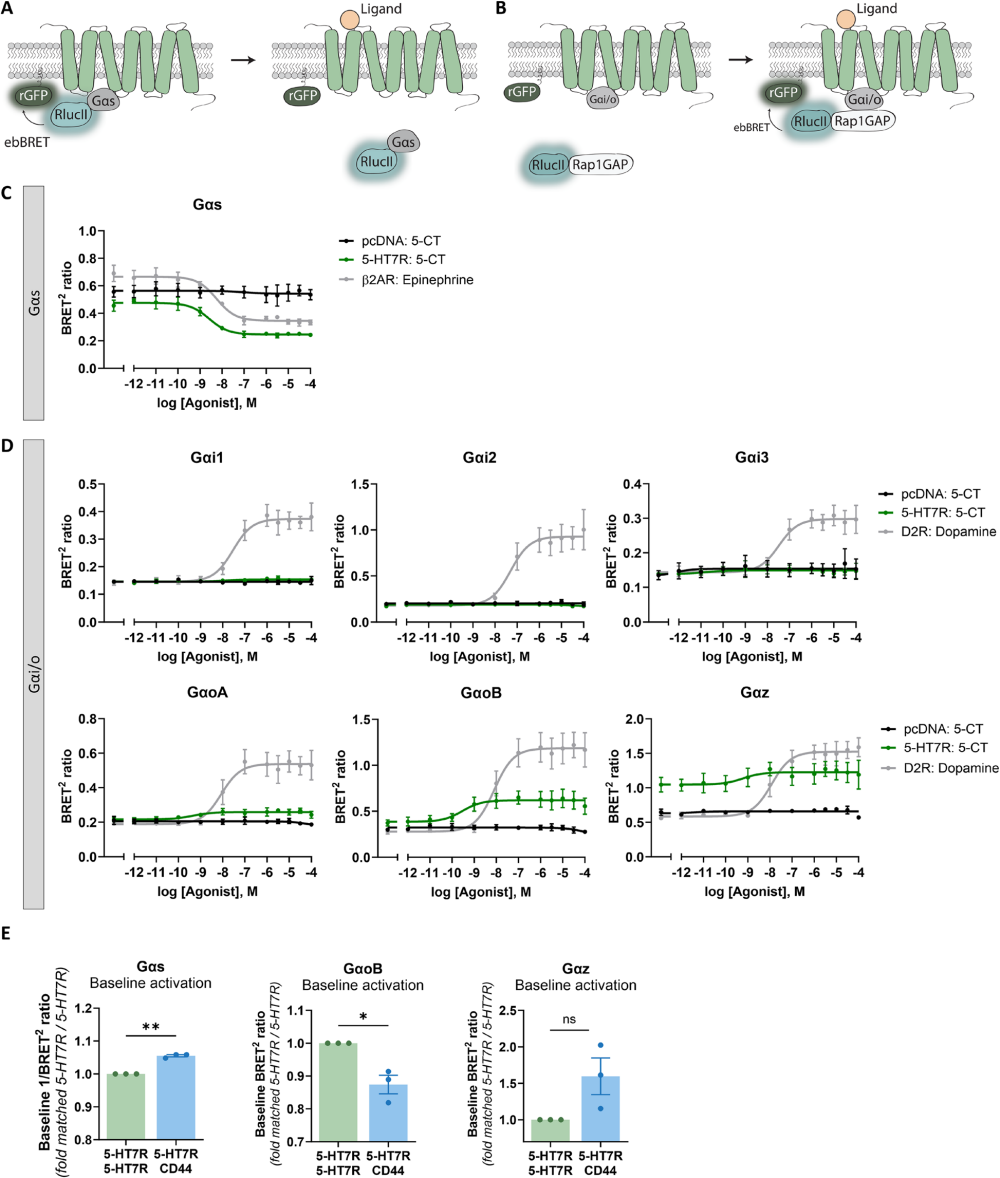
5-HT7R-mediated activation of Gα proteins is distinctly modified in the presence of CD44. **A**-**B** Schema of the BRET^2^-based G protein Effector Membrane Translocation Assay or GEMTA [40] that was used to study Gα protein activation. Upon activation, RlucII-linked Gαs leaves the plasma membrane, which results in a decreased enhanced bystander BRET (ebBRET) signal between the RlucII and the membrane-anchored rGFP-CAAX (**A**). Activation of Gαi/o proteins lead to the recruitment of RlucII-tagged Rap1GAP leading to an increased ebBRET (**B**). **C**-**D** Dose–response curves of the BRET^2^ ratio upon treatment with increasing concentrations of 5-CT in HEK-293 cells expressing pcDNA (grey), 5-HT7R (green), or a positive control (black) combined with the Gαs (**C**) or Gαi/o family (**D**) specific biosensor components (see Methods section). Cells expressing β2 adrenergic receptor (β2AR) or dopamine D2 receptor (D2R) were treated with epinephrine or dopamine, respectively. Data are means ± SEM (*N* = 3 experiment days). **E** Baseline BRET^2^ ratios estimated for HEK-293 cells expressing the 5-HT7R alone or in combination with CD44 assessed with the Gαs, or GαoB, or Gαz specific biosensors. Values are calculated relative to 5-HT7R/5-HT7R. Data are means ± SEM (*N* = 3 experiment days). Statistical significance was assessed by one sample t-test. * *p* < 0.05, ** *p* < 0.01, ns: not significant

Having shown that 5-HT7R-CD44 heteromerization modulates 5-HT7R-mediated cAMP production, we wondered whether this effect comes from altered coupling and/or activation of heterotrimeric G proteins. Therefore, we next investigated the activation of different Gα protein subtypes known to modulate cAMP production, including Gαs (cAMP increase) and members of the Gαi/o family (cAMP decrease) [49, 50]. We utilized the previously established G protein effector membrane translocation assay (GEMTA) [40], which is based on the detection of bioluminescence resonance energy transfer (BRET) between the luciferase RlucII and membrane-anchored rGFP from *Renilla reformis*. In the case of Gαs, the RlucII is coupled directly to the Gα protein. Upon activation of Gαs, the Gα subunit leaves the plasma membrane, which results in a decrease of the BRET ratio (Fig. 6A). In contrast, activation of Gα subunits from the Gαi/o family results in recruitment of the RlucII-tagged Gαi/o-specific effector Rap1GAP to the plasma membrane (Fig. 6B).

First, we defined which of the Gα proteins modulating cAMP levels (i.e. Gαs, Gαi1, Gαi2, Gαi3, GαoA, GαoB, Gαz) can be activated by the 5-HT7R. To this end, we measured BRET ratio in HEK-293 cells co-expressing the 5-HT7R and the G protein (see Methods section) upon treatment with increasing concentration of 5-CT. Functionality of the assay was verified using Gαs-coupled β2 adrenergic receptor stimulated with epinephrine, and the Gαi/o-coupled dopamine D2 receptor stimulated with dopamine (Fig. 6C and D). As shown in Fig. 6C, we found dose-dependent activation of Gαs by 5-HT7R (logEC_50_ = -8.59 ± 0.19). While Gαi1, Gαi2, Gαi3, and GαoA were not activated by the 5-HT7R, we observed 5-HT7R-mediated activation of GαoB (logEC_50_ = -9.54 ± 0.55) and Gαz (logEC_50_ = -9.22 ± 1.18; Fig. 6D).

One of our main findings from the cAMP measurements was an increased 5-HT7R-mediated basal cAMP level in the presence of CD44. Therefore, we next investigated baseline activation of the three aforementioned Gα proteins by 5-HT7R in the absence and presence of CD44 (Fig. 6E, Supplementary Fig. S7A-C). In the case of the Gαs protein, we found a significant increase in its baseline activation upon CD44 co-expression. In contrast, heteromerization leads to a significant decrease of GαoB activation, while baseline activation of Gαz was not affected (Fig. 6E). When we compared 5-CT-mediated G protein responses, we did not observed any significant differences in the maximal response amplitude between 5-HT7R-5-HT7R homomers and 5-HT7R-CD44 heteromers (Supplementary Fig. S7).

An interesting finding of the present study is the observed transactivation of the 5-HT7R upon stimulation of CD44 in the heteromeric 5-HT7R/CD44 complexes. As the 5-HT7R can activate Gαs and members of the Gαi/o family, we investigated whether the observed transactivation process is mediated by an activation of Gαs or an inhibition of Gαi/o. Therefore, Gαi/o activity was blocked using pertussis toxin (PTX) prior to the stimulation with hyaluronic acid and 5-HT7R activation was assessed by cAMP measurements (Supplementary Fig. S8). Interestingly, pre-treatment with PTX reduced but did not completely abolish the hyaluronic acid-induced cAMP production, suggesting a combined involvement of Gαs and Gαi/o in the transactivation process.

### Expression of 5-HT7R and CD44 in the brain changes during development

Finally, we analyzed whether the relative concentration of 5-HT7R-CD44 heteromers, which depends on the expression ratio of both receptors, undergoes developmental changes in the brain. For that, we determined the expression profiles for 5-HT7R and CD44 in the mouse hippocampus (HIP) and prefrontal cortex (PFC) at different stages of postnatal development using real-time quantitative PCR. This approach demonstrated that the highest amount of 5-HT7R transcripts in both analyzed brain regions was found during early postnatal stage (postnatal day 5 or P5) and downregulated during later developmental stages (Supplementary Fig. S9A and D). A similar expression profile was observed for CD44 mRNA transcripts (Supplementary Fig. S9B and E). Because the protein expression level is assumed to roughly correlate with the level of mRNA transcripts, the above data suggest that receptor expression also undergoes developmental regulation. Such differences in the expression levels result in significant changes of the 5-HT7R to CD44 ratio from 1:1.5 at P15 to 2:1 at 12 months in the PFC, and from 1:2.5 at P15 to 2:1 at 22 months in the HIP (Supplementary Fig. S9C and F). In combination with our dimerization model (Fig. 1F), which suggests similar dissociation constants for homo- and heteromers, one can hypothesize that the amount of 5-HT7R/CD44 oligomers and their functional roles might change during brain development.

## Discussion

**Fig. 7:**
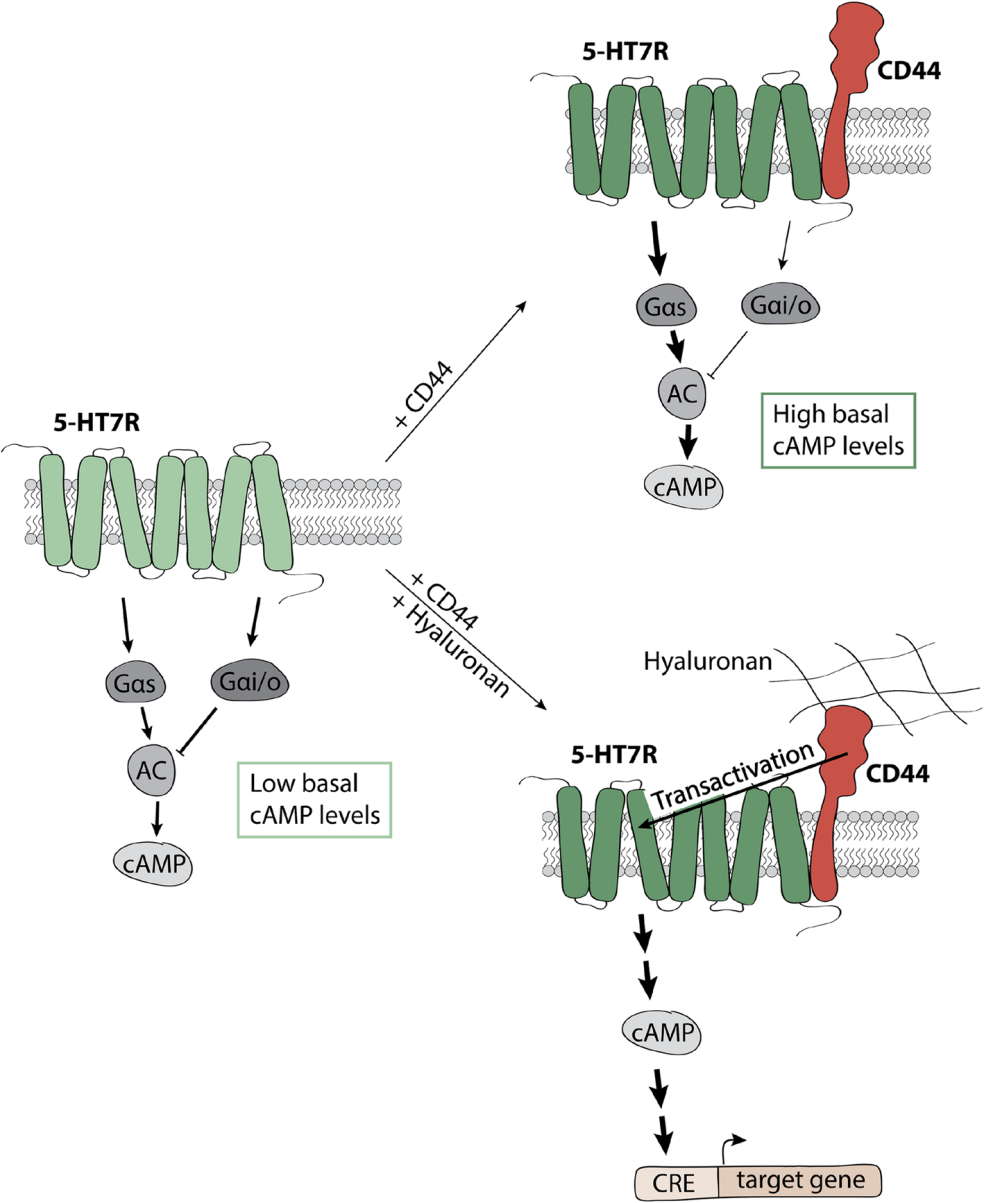
Schematic overview of the effects of CD44 co-expression on 5-HT7R-mediated signaling. Upon the formation of heteromeric complexes with CD44, the constitutive activity of the 5-HT7R towards cAMP signaling is increased, whereas the agonist-induced activity is decreased. The modulated constitutive activity results from an increased Gαs and a decreased GαoB activation under basal conditions (upper panel). Stimulation of heteromeric complexes with the CD44 ligand hyaluronic acid leads to the 5-HT7R-mediated production of cAMP and the subsequent activation of CRE-mediated gene expression (lower panel)

Over the last decades, it has been demonstrated that GPCR signaling can be influenced by oligomerization with other receptors [3–6]. In the present study, we confirmed a physical interaction between the serotonin receptor 5-HT7R, a class A GPCR, and the hyaluronan receptor CD44, a single-pass transmembrane cell adhesion molecule. We also demonstrated that interaction with CD44 modulates the 5-HT7R-mediated cAMP signaling by increasing the constitutive activity and by reducing the agonist-induced activation of the 5-HT7R (Fig. 7). CD44 has previously been shown to act as an allosteric modulator or co-receptor for several non-GPCRs, including the receptor tyrosine kinase c-Met and the purinergic P2X7 receptor [51, 52]. Only one study has investigated the influence of the interaction of CD44 on GPCR-mediated signaling, which demonstrated an interaction between CD44 and the chemokine receptor CXCR4, a class A GPCR [53]. In that study, binding of high-molecular weight hyaluronan to CD44 facilitated CXCL12-mediated activation, demonstrating that interaction with CD44 can modulate GPCR-mediated signaling. In general, modulatory interactions between GPCRs and the extracellular matrix (ECM) have been mainly described for secreted ECM proteins, including interaction between GABA_B_ receptor and Cartilage oligomeric matrix protein and AT1 receptor and Fibulin-2 [54–56]. Moreover, heteromeric complexes between PECAM-1 (platelet-endothelial cell adhesion molecule-1), a transmembrane cell adhesion molecule, and the bradykinin receptor B2 (BKRB2), a GPCR, have been described [57].

In our previous study, we have demonstrated that the 5-HT7R predominantly forms homodimers [7]. CD44 was also suggested to primarily form dimers [43, 58, 59]. In general, it is widely accepted that GPCRs can form dimers as well as higher-order oligomers, depending on the interaction partners. In contrast, oligomerization between GPCRs and non-GPCRs have been much less studied. Consequently, far less is known about the stoichiometry of such complexes. For example, interactions between GPCRs and the single-transmembrane-spanning receptor-activity modifying proteins (RAMPs) have been discussed to be either dimeric, trimeric or tetrameric, making it difficult to predict the exact stoichiometry [60–62]. Therefore, additional studies would be needed to define the exact stoichiometry of the 5-HT7R/CD44 complexes.

Accumulating evidence demonstrated that multiple GPCRs exhibit an agonist-independent constitutive activity, which plays an important functional role [18–22, 63]. Among others, constitutive activity can be influenced by the GPCR’s dimerization state, as it has been demonstrated for the β2AR homodimers and CXCR4/CXCR heterodimers [64, 65]. According to the two-state model, a GPCR may exist in an equilibrium between its active and its inactive states, undergoing different conformational changes when switching between both states. In case of 5-HT7R-CD44 heteromerization, interaction with CD44 might either shift this equilibrium towards the 5-HT7R active conformation or stabilize such a conformation, leading to increased agonist-independent cAMP production. This will result in a higher proportion of already activated 5-HT7R under basal conditions, which can explain the reduced agonist-induced response observed in the presence of CD44. Searching for signaling mechanisms contributing to the altered cAMP signaling, we found that 5-HT7R not only couples to the Gαs but also to GαoB and Gαz, which both belong to the Gαi/o family. Similar couplings of 5HT7R to Gi/o family members has previously been reported [66, 67] and could represent a mechanism for tight regulation of 5-HT7R-mediated cAMP signaling. Indeed, our data suggest that the elevated basal cAMP levels obtained for heteromers may be a result of simultaneously increased Gαs and decreased GαoB activation in the presence of CD44. Changes in G protein selectivity upon oligomerization has been described for several GPCRs. This includes the CCR2/CXCR5 heterodimer, the platelet-activating factor receptor (PAFR) homodimers, and the µ-opioid/δ-opioid receptor heterodimers [68–70]. When expressing the µ-opioid receptor alone, GH3 cells show inhibited spontaneous Ca^2+^ signals, which is mediated by the activation of different pathways, including the inhibition of the adenylate cyclase. Upon co-expression of the δ-opioid receptor, cells showed an increased Ca^2+^ signaling. This change in signaling properties was suggested to result from a switch in G protein selectivity from Gi to Gq [68]. In contrast, homodimerization of the PFAR seems to lead to an increased Gq signaling, while β-arrestin recruitment is reduced [69]. Moreover, CCR2 and CCR5 heterodimers interact with the Gq/11 protein, while such coupling cannot be detected when one of the receptors was expressed alone [70].

One of the main findings of our study is the observation that activation of CD44 within the heteromeric complex with its natural ligand hyaluronan boosts constitutive 5-HT7R-mediated cAMP production under basal conditions highlighting a novel transactivation mechanism for 5-HT7R. Since this effect was evident even for the activation of the CRE-mediated transcription, 5-HT7R transactivation by CD44 may thus represent a novel mechanism for regulating 5-HT7R-mediated gene expression (Fig. 7). The secretion of hyaluronan is tightly regulated during neuronal development as well as under several pathological conditions. For example, increased levels of hyaluronic acid have been found in the cerebrospinal fluid and the temporal cortex of Alzheimer`s disease patients [71, 72]. Interestingly, after traumatic brain injury, the expression of hyaluronidases and CD44 was increased in rats [73]. During rat brain development it was found that the amount of hyaluronic acid peaked at postnatal day seven and then decreased rapidly during the next two weeks until it reached the levels in the adult brain [74]. Since hyaluronan represents the main component in the brain ECM, even small variations in its amount or composition might have a prominent impact on 5-HT7R-mediated signaling via the proposed transactivation mechanism.

The 5-HT7R plays a pivotal role in the regulation of various fundamental brain functions, including mood control, learning and memory processing, as well as regulation of the circadian rhythm. Therefore, tight regulation of receptor-mediated signaling is crucial for proper brain function, and a dysregulation of signaling can result in different brain pathologies [11, 12, 15]. As we demonstrated here, heteromerization with CD44 mainly influenced constitutive 5-HT7R activity leading to increased basal cAMP levels. In the brain, cAMP is crucial for many neuronal processes. For example, cAMP has been found to regulate brain development, neuronal excitability, synaptic plasticity, as well as learning and memory [75]. The involvement of the cAMP-CREB-CRE pathway in learning and memory has been first described for the *Aplysia*, where injection of the CRE sequence negatively affects long-term synaptic strength [76]. Several studies suggest that CRE-driven gene transcription is involved in a long-term potentiation as well as hippocampus-dependent memory [77–79]. Also in the medial prefrontal cortex, anterior cingulate cortex, and the amygdala the cAMP-CREB-CRE pathway can be linked to memory-related functions [80, 81]. A more recent study demonstrated that reducing cAMP or PKA activity during early development abolished the formation of functional synapses [82]. Genes controlled by CRE pathway include proteins involved in neurotransmission, growth factors, metabolic enzymes, as well as other transcription factors and proteins involved in signal transduction [83]. Therefore, altered 5-HT7R signaling by heteromerization might influence these processes via modulating cAMP levels. Supporting this view, a recently published study demonstrated that 5-HT7R-mediated cAMP signaling is important for spine maturation in the prefrontal cortex in particular during the early brain development [84].

Our results demonstrated that the expression levels of 5-HT7R and CD44 undergo pronounced changes in the cerebral cortex and hippocampus during the development: Both receptors are highly expressed at early postnatal stages and their amounts progressively decrease during development, although at different rates. Therefore, the relative concentration of 5-HT7R-CD44 heteromers and, as a consequence, their functional importance will also undergo pronounced developmental changes. A relative high expression level of 5-HT7R-CD44 heteromers at the early postnatal stages will result in higher basal cAMP level, which can be further increased by the hyaluronic acid-mediated 5-HT7R transactivation. With increasing age, the relative amount of heteromers will gradually decrease, allowing 5-HT7R homomers to become the dominant population. Thus, the stimulatory influence of heteromers on both basal and hyaluronic acid-mediated cAMP production begins to subside, while serotonin-mediated 5-HT7R-mediated signaling becomes more prominent.

## Conclusions

In the present study, we demonstrated that the class A GPCR 5-HT7R and the hyaluronan receptor CD44 form homo- and heteromeric complexes with the similar efficiency. Heteromerization results in an increased constitutive and decreased agonist-induced activity of the 5-HT7R towards cAMP production. Mechanistically, heteromerization leads to a decrease of 5-HT7R-mediated GαoB and increase of Gαs protein activation. More importantly, stimulation of CD44 within the 5-HT7R-CD44 complexes with its natural ligand hyaluronic acid results in strong 5-HT7R transactivation towards cAMP response and CRE-mediated transcription. Since the 5-HT7R-mediated signaling plays a pivotal role in many physiological as well as pathological processes [11, 12, 15, 75], interaction with CD44 might represent a novel molecular mechanism regulating 5-HT7R-mediated signaling. Taken together, our study suggests that the regulated and balanced ratio of 5-HT7R-CD44 heteromerization may be critically involved in the precise modulation of 5-HT7R functions during different physiological and pathological processes in the brain.

## Supplementary Information

Supplementary Material 1.

## Data Availability

No datasets were generated or analysed during the current study.

## Abbreviations

β2AR: β2 Adrenergic receptor
5-CT: 5-Carboxamidotryptamine
5-HT: Serotonin
5-HT7R: Serotonin receptor 7
A: Acceptor
ANOVA: Analysis of variance
BRET: Bioluminescence resonance energy transfer
CMV: Cytomegalovirus
CRE: cAMP response element
CREB: cAMP response element binding protein
D: Donor
DMEM: Dulbecco´s modified Eagles medium
ebBRET: Enhanced bystander BRET
eCFP: Enhanced cyan fluorescent protein
eGFP: Enhanced green fluorescent protein
ECM: Extracellular matrix
eYFP: Enhanced yellow fluorescent protein
FCS: Fetal calf serum
FRET: Förster resonance energy transfer
GPCR: G protein-coupled receptor
HA: Hemagglutinin
HIP: Hippocampus
IBMX: 3-Isobutyl-1-methylxanthin
lux-FRET: Linear unmixing FRET
MMP-9: Matrix metalloproteinase-9
NCS: Newborn calf serum
PCR: Polymerase chain reaction
PFC: Prefrontal cortex
PKA: Protein kinase A
rGFP: *Renilla* Green fluorescent proteins
RTK: Receptor tyrosine kinase
SEM: Standard error of the mean
TEV: Tobacco etch virus
