# Supplementary material for: The cell adhesion molecule CD44 acts as a modulator of 5-HT7 receptor functions
Source: Cell Commun Signal. 2024 Nov 23;22:563. doi: 10.1186/s12964-024-01931-0 (PMC11585102; doi:10.1186/s12964-024-01931-0)
Supplement: Supplementary file 1 — Supplementary Material 1. [file 12964_2024_1931_MOESM1_ESM.pdf]

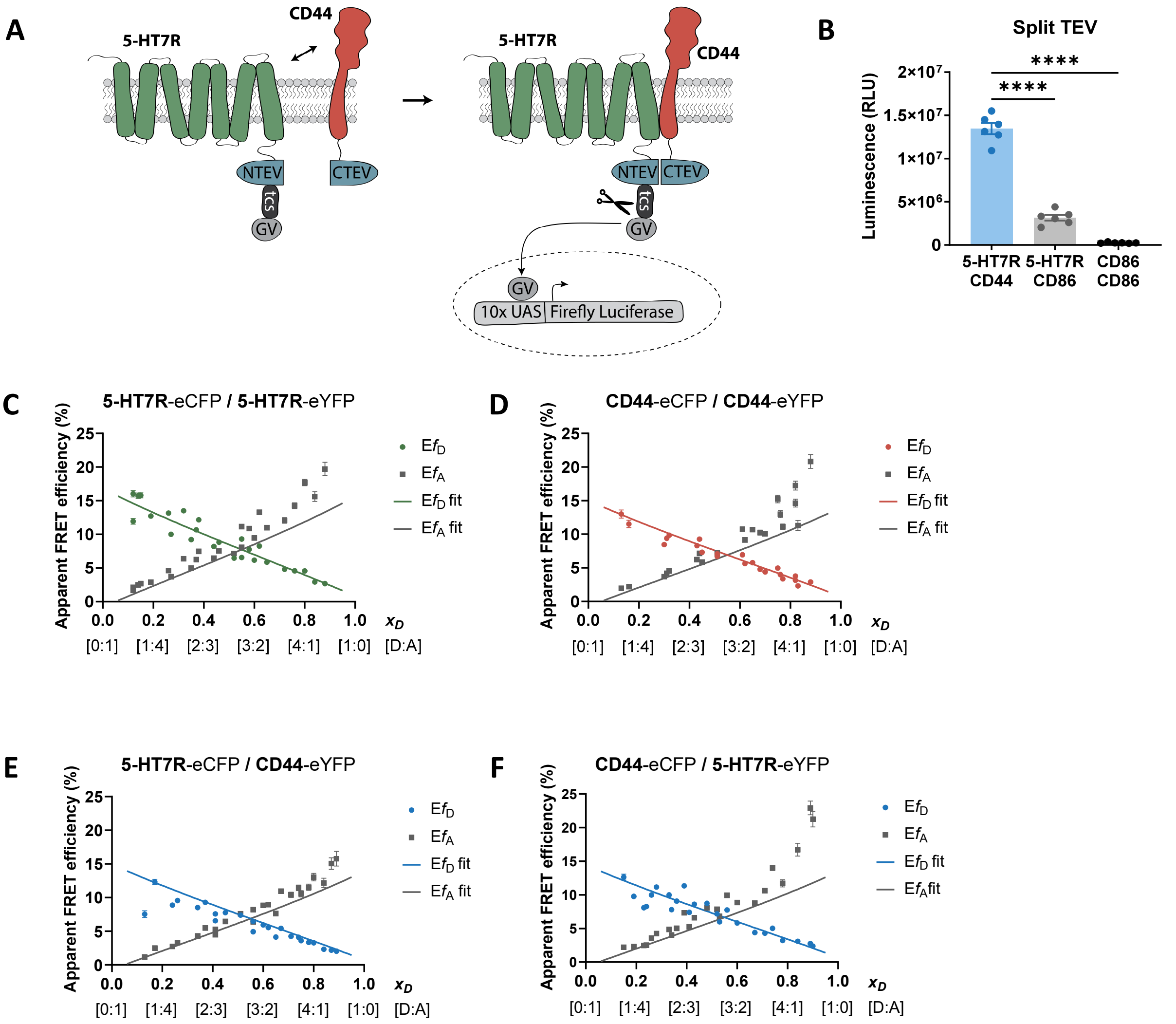

Supplementary Figure S1: Investigation of 5-HT7R and CD44 heteromerization by Split TEV and lux-FRET.

A: Schema of the Split-TEV assay. Receptors of interest are linked to either the N-terminal or C-terminal fraction of the NIa protease from the tobacco etch virus (TEV). NTEV constructs are additionally linked to a TEV cleavage site (tcs) and the transcriptional coactivator GAL4-VP16 (GV). Upon dimerization, the TEV constructs are complemented and the protease is activated. After cleavage of the tcs, the GV translocates into the nucleus where it binds to the upstream activated sequences (10x UAS) and induces expression of a luciferase construct.

B: Split-TEV assay in N1E-115 cells. Luminescence was measured from cells expressing 5-HT7R-NTEV/CD44-CTEV, 5-HT7R-NTEV/CD86-CTEV, or CD86-NTEV/CD86-CTEV. Data are means ± SEM (N = 6). Statistical significance was assessed by one-way ANOVA with post hoc Dunnett's multiple comparison test. **** $p < 0.0001$.

C-F: Apparent FRET efficiencies E*f*D (colored) and E*f*A (grey) were plotted as a function of the donor mole fraction x_D for 5-HT7R-homomers (**C**), CD44-homomers (**D**), and 5-HT7R/CD44-heteromers (**E**, **F**). Fitting of the experimental data was used to estimate the relative dissociation constants of the homo- and heterodimerization processes. Error of the fit: χ^2 = 0.0008 (N = 3 experiment days, 6 measurements).

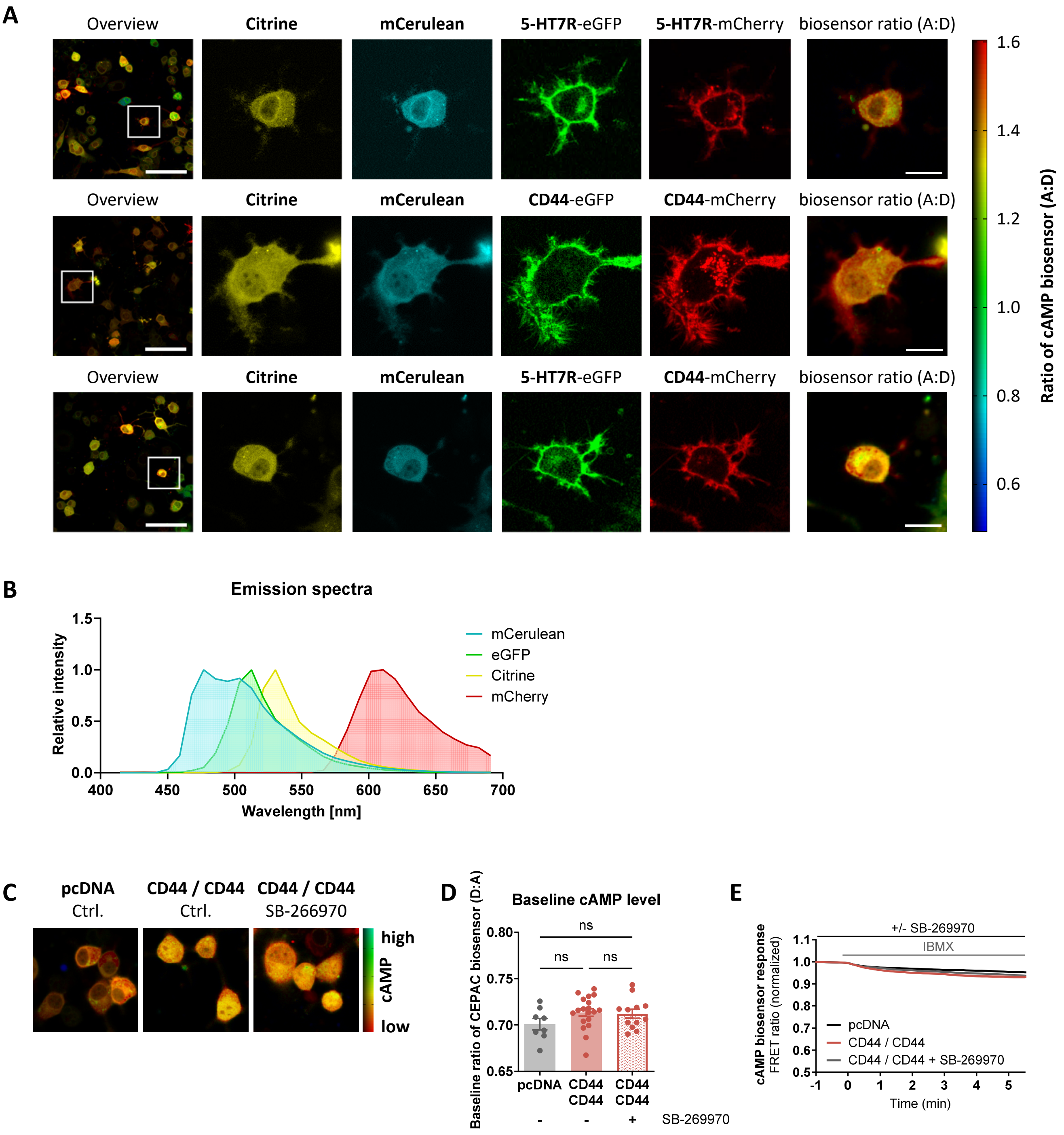

Supplementary Figure S2: cAMP measurements in N1E-115 cells.

A: Representative overview and single cell images of N1E-115 cells transiently co-expressing the cAMP biosensor (Citrine: yellow, mCerulean: cyan) and 5-HT7R-eGFP/5-HT7R-mCherry, CD44-eGFP/CD44-mCherry, or 5-HT7R-eGFP/CD44-mCherry (eGFP: green, mCherry: red). The right images show the acceptor/donor (A/D, Citrine/mCerulean) ratio of the CEPAC biosensor. Scale bar: 100 µm, inset scale bar: 20 µm.

B: Fluorescence emission spectra of N1E-115 cells expressing mCerulean (blue), 5-HT7R-eGFP (green), Citrine (yellow), and CD44-mCherry (red). Intensity values were normalized to the maximum intensity of each spectrum.

C, D: Representative images (**C**) and quantification (**D**) of the baseline cAMP biosensor ratio in N1E-115 cells expressing pcDNA or CD44-eGFP/CD44-mCherry. Cells were pre-treated as indicated. Data are means ± SEM ($N \geq 8$ experiment days, ≥ 23 coverslips, ≥ 573 cells). Statistical significance was assessed by one-way ANOVA. ns: not significant

E: Time-response curves of the cAMP biosensor in response to treatment with IBMX in N1E-115 cells transiently expressing pcDNA or CD44-eGFP/CD44-mCherry. Cells were pre-treated as indicated. Data are means ± SEM ($N \geq 3$ experiment days, ≥ 6 coverslips, ≥ 119 cells).

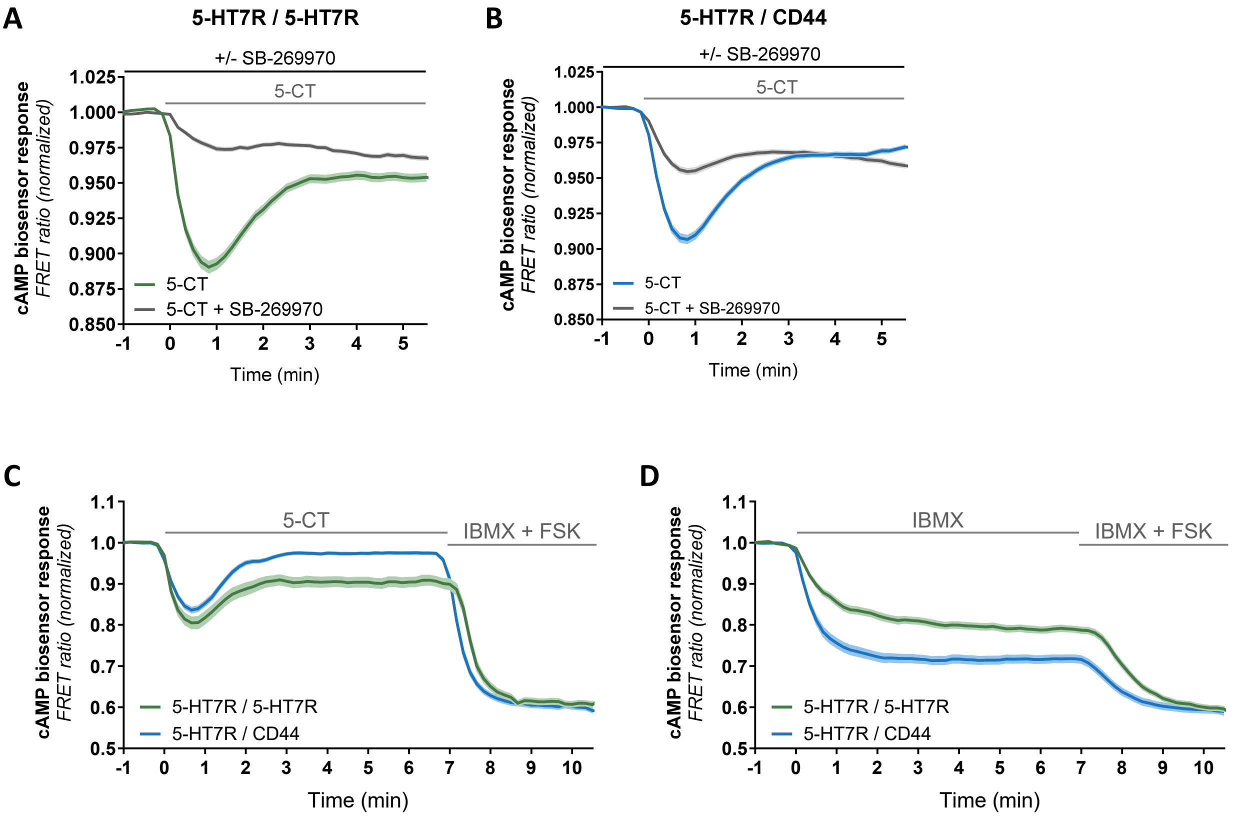

Supplementary Figure S3: 5-CT-induced 5-HT7R-mediated cAMP response can be blocked by SB-269970.

A, B: Traces showing changes in the fluorescence ratio of the cAMP in response to 5-CT alone or in combination with SB-269970 in N1E-115 cells expressing 5-HT7R-eGFP/5-HT7R-mCherry (**A**) or 5-HT7R-eGFP/CD44-mCherry (**B**). Baseline ratios were normalized to one. Data are means ± SEM (N = 6 experiment days, ≥ 10 coverslips, ≥ 181 cells).

C, D: Representative traces of the cAMP biosensor ratio. N1E-115 cells expressing either 5-HT7R-eGFP/5-HT7R-mCherry or 5-HT7R-eGFP/CD44-mCherry were first treated with IBMX (**C**) or 5-CT (**D**) and afterwards with IBMX and forskolin (FSK) simultaneously. Data are means ± SEM (N = 2).

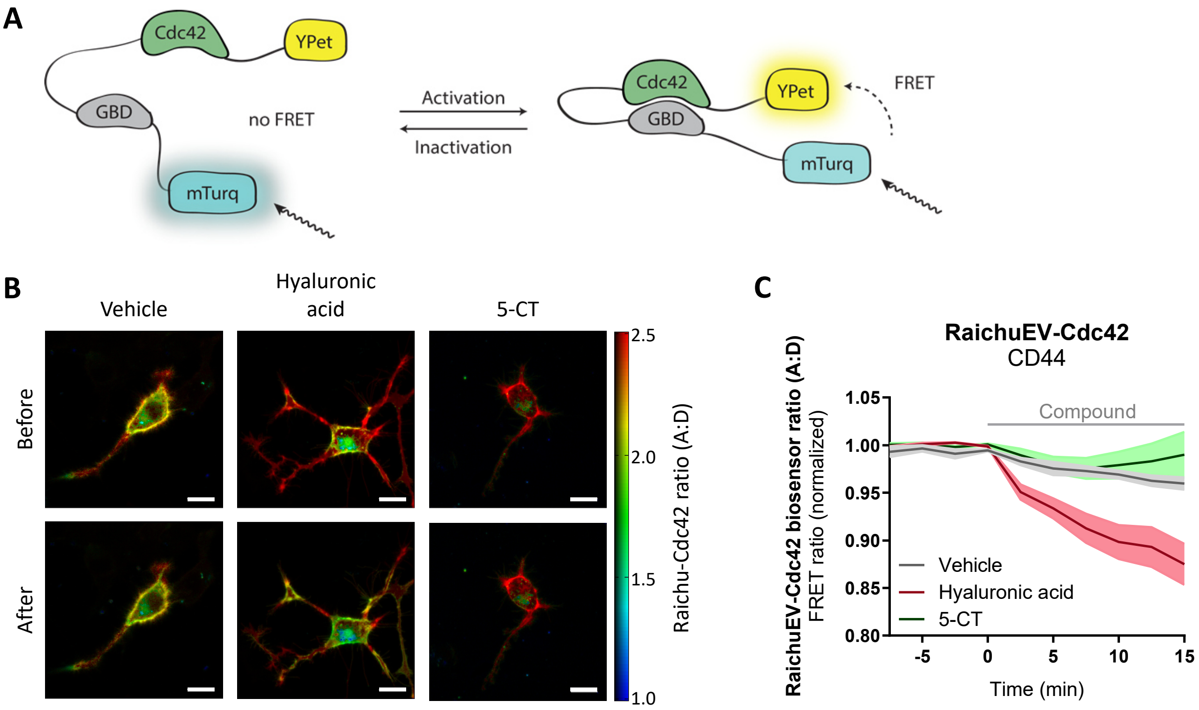

Supplementary Figure S4: Stimulation of CD44 results in inhibition of Cdc42 correlating with an activation of CD44.

A: Schematic representation of the FRET-based Raichu-Cdc42 biosensor consisting of the fluorophores mTurquoise (mTurq) and YPet, the GTPase-binding domain (GBD) and the small GTPase Cdc42. Activation of Cdc42 correlates with an increase in the YPet:mTurq (acceptor:donor, A:D) ratio.

B: Representative images demonstrating the A:D ratio of the Raichu-Cdc42 biosensor in N1E-115 cells expressing CD44-mCherry before and after stimulation with vehicle, hyaluronic acid (50 µg/mL), or 5-CT (10 µM). Scale bar: 20 µm.

C: Changes in A:D ratio of the Raichu-Cdc42 biosensor upon stimulation of N1E-115 cells expressing CD44-mCherry. Baseline ratios were normalized to one. Data are means ± SEM (N ≥ 5 coverslips).

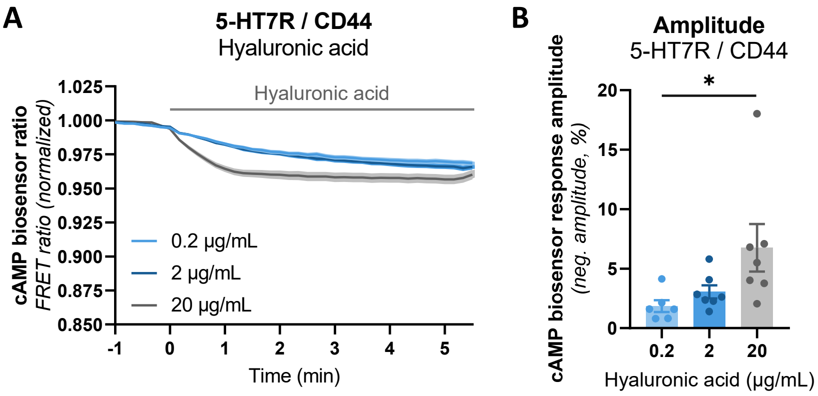

Supplementary Figure S5: Transactivation of 5-HT7R via stimulation of CD44 by hyaluronic acid is dose-dependent.

A: Changes in the cAMP biosensor ratio upon stimulation of N1E-115 cells expressing 5-HT7R-eGFP and CD44-mCherry with increasing concentrations of hyaluronic acid. Baseline ratios were normalized to one. Data are means ± SEM ($N \geq 6$ biological replicates, ≥ 6 coverslips, ≥ 146 cells).

B: Response amplitudes were calculated from the respective response curves from (**A**). Data are means ± SEM ($N \geq 6$ biological replicates, ≥ 6 coverslips, ≥ 146 cells). Outliers were identified using the robust regression and outlier removal ($Q = 1\%$). Statistical significance was assessed by one-way ANOVA with post hoc Tukey's multiple comparison test. *$p < 0.05$.

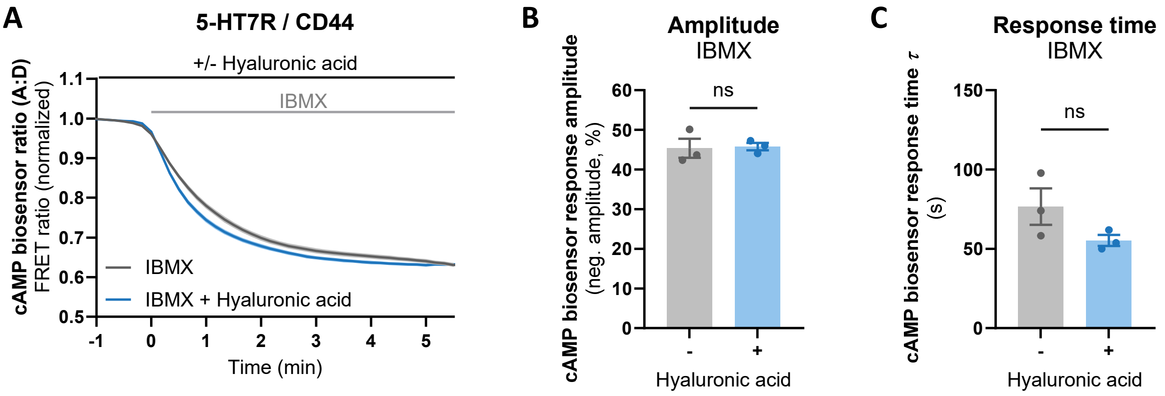

Supplementary Figure S6: Stimulation of CD44 in hetero-oligomeric 5-HT7R/CD44 complexes with hyaluronic acid does not affect 5-HT7R constitutive activity.

A: Traces showing the changes in the cAMP biosensor ratio in N1E-115 cells after application of the PDE inhibitor IBMX with or without pre-treatment with hyaluronic acid. Cells were expressing 5-HT7R-eGFP and CD44-mCherry. Baseline values were normalized to one. Data are means ± SEM (N = 3 biological replicates, 3 coverslips, ≥ 195 cells).

B, **C**: Response amplitude (**B**) and response time (**C**) calculated from the respective traces in (**A**). Data are means ± SEM (N = 3 biological replicates, 3 coverslips, ≥ 195 cells). Statistical significance was assessed using unpaired t-test. Ns: not significant.

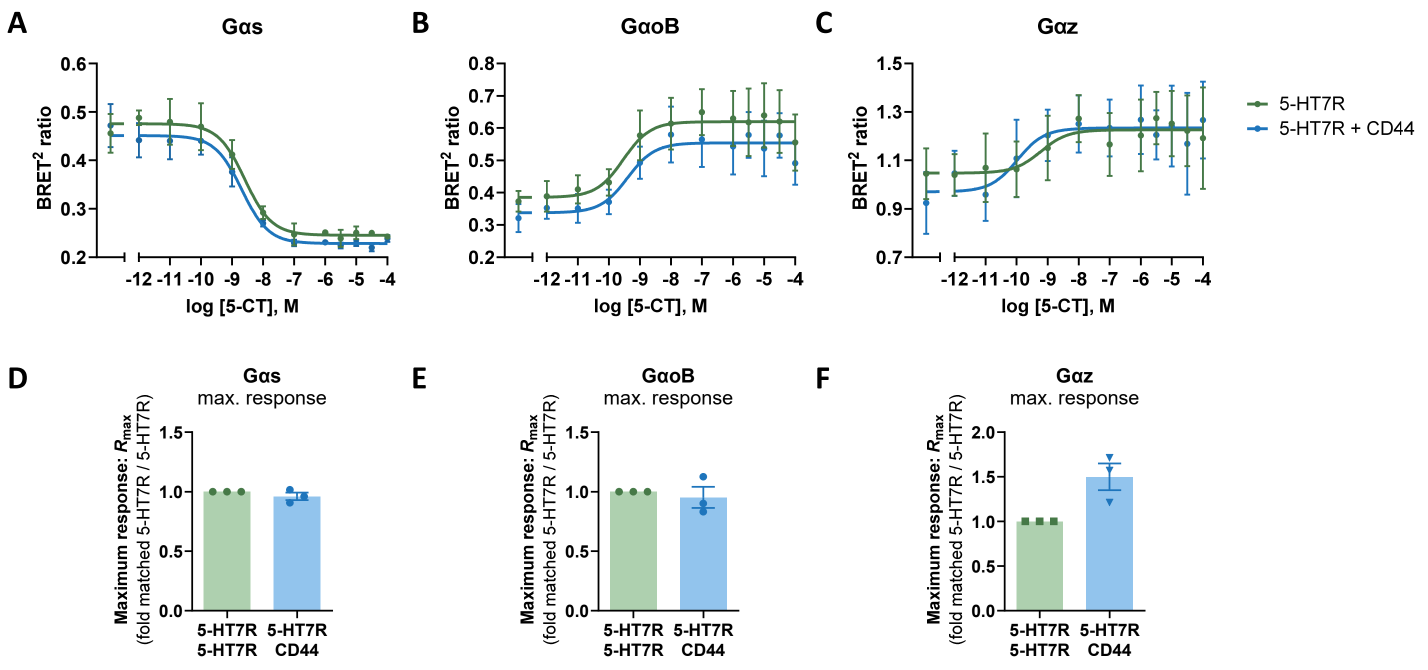

Supplementary Figure S7: Dose-response curves of Gα protein activation after 5-CT stimulation.

A-C: Dose-response curves of $BRET^2$ ratio upon increasing concentrations of 5-CT in HEK-293 cells expressing 5-HT7R alone (grey) or together with CD44 (blue) combined with the Gαs (**A**), GαoB (**B**), or Gαz (**C**) specific biosensor components (see *Methods* section). Calculated minimum values were used as baseline $BRET^2$ ratios (depicted in Figure 6 **E**). Data are means ± SEM (N = 3 experiment days).

D-F: Response amplitudes of activation of Gαs (**D**), GαoB (**E**), or Gαz (**F**) after stimulation of the 5-HT7R with 5-CT in the presence or absence of CD44. Values were obtained from the dose-response curves in (**A**), (**B**), and (**C**), respectively. Data are means ± SEM (N = 3 experiment days). Statistical significance was assessed by one-sample t-test. $p > 0.05$.

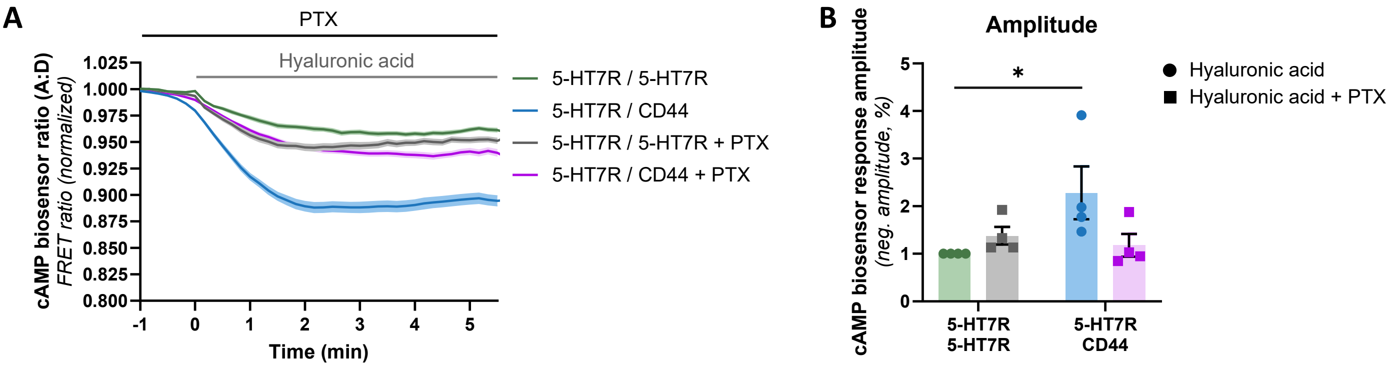

Supplementary Figure S8: Pre-inhibition of Gi/o proteins reduces but does not abolish transactivation of 5-HT7R via stimulation of 5-HT7R/CD44 heteromeric complexes with hyaluronic acid.

Time-response curves (**A**) and amplitudes (**B**) showing the changes in the cAMP biosensor ratio upon stimulation of N1E-115 cells with hyaluronic acid in the absence or presence of the Gi/o inhibitor pertussis toxin (PTX). Cells were expressing either 5-HT7R-eGFP/5-HT7R-mCherry or 5-HT7R-eGFP/CD44-mCherry. Baseline ratios were normalized to one. Data are means ± SEM (N = 4 biological replicates, = 4 coverslips, ≥ 128 cells). Statistical significance was assessed using Kruskal-Wallis test with Dunn's multiple comparisons test. *$p < 0.05$.

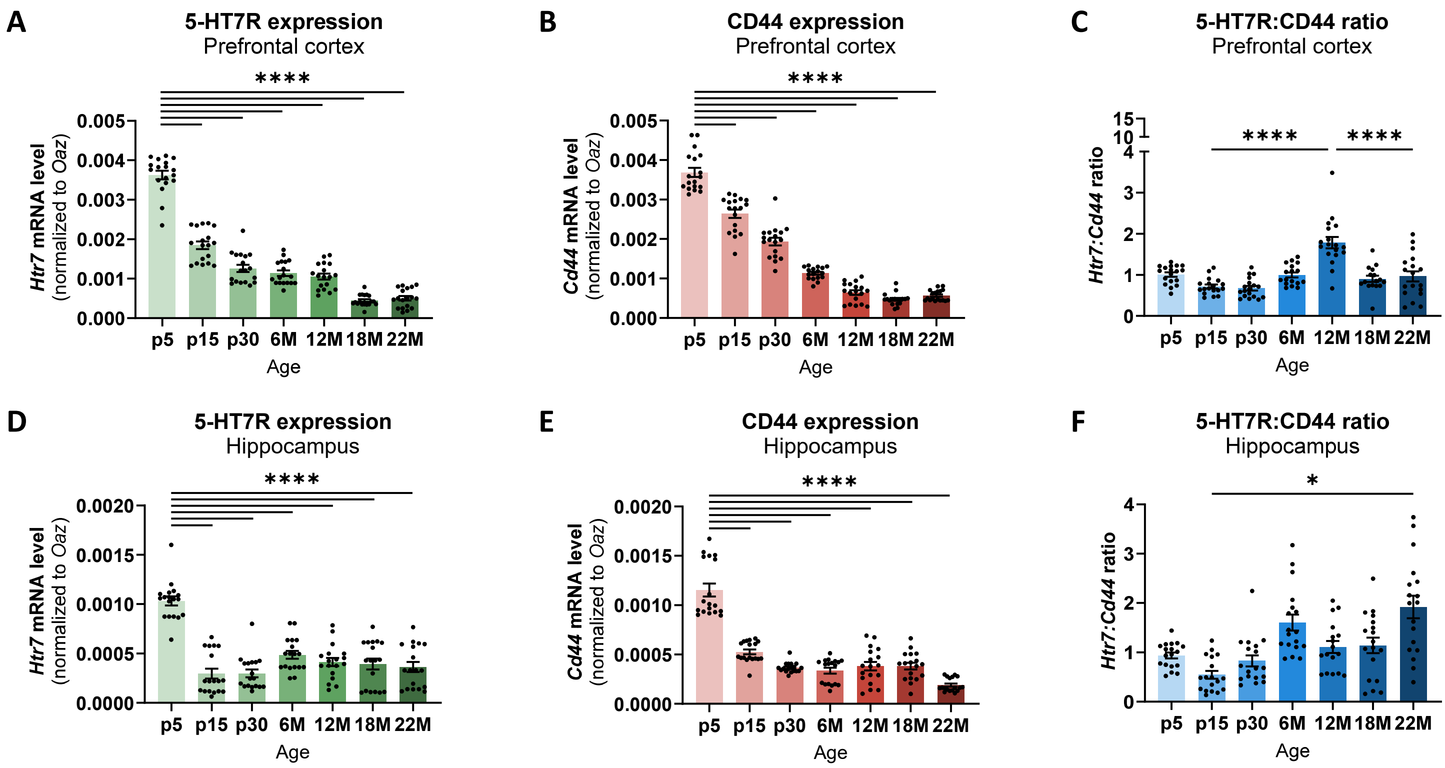

Supplementary Figure S9: Expression levels of 5-HT7R and CD44 change during brain development.

A, B, D, E: Expression levels of mRNA encoding 5-HT7R (**A**, **D**) or CD44 (**B**, **E**) in the prefrontal cortex (PFC) and the hippocampus (HIP) as indicated. mRNA levels were determined in mouse brains at different postnatal days (p) and months (M) by qRT-PCR. Presented is the expression normalized to *Oaz*, which was used as a calibration control. Data are means ± SEM (N ≥ 17 mice). Outliers were identified using the robust regression and outlier removal (Q = 1%). Statistical significance was assessed by one-way ANOVA with post hoc Dunnett's multiple comparison test (comparison to p5). **** p < 0.0001.

C, F: Ratio of 5-HT7R to CD44 mRNA expression levels in the indicated brain regions. Data are means ± SEM (N ≥ 16 mice). Outliers were identified using the robust regression and outlier removal (Q = 1%). Statistical significance was assessed by one-way ANOVA with post hoc Tukey's multiple comparison test (PFC: p15 vs 12M, 12M vs 22M; HIP: p15 vs 22M). * p < 0.05, **** p < 0.0001.
